# World Allergy Organization-McMaster University Guidelines for Allergic Disease Prevention (GLAD-P): Vitamin D

**DOI:** 10.1186/s40413-016-0108-1

**Published:** 2016-05-17

**Authors:** Juan José Yepes-Nuñez, Alessandro Fiocchi, Ruby Pawankar, Carlos A. Cuello-Garcia, Yuan Zhang, Gian Paolo Morgano, Kangmo Ahn, Suleiman Al-Hammadi, Arnav Agarwal, Shreyas Gandhi, Kirsten Beyer, Wesley Burks, Giorgio W. Canonica, Motohiro Ebisawa, Rose Kamenwa, Bee Wah Lee, Haiqi Li, Susan Prescott, John J. Riva, Lanny Rosenwasser, Hugh Sampson, Michael Spigler, Luigi Terracciano, Andrea Vereda, Susan Waserman, Holger J. Schünemann, Jan L. Brożek

**Affiliations:** Department of Clinical Epidemiology & Biostatistics, McMaster University Health Sciences Centre, Room 2C16 1280 Main Street West, Hamilton, L8N 4K1 ON Canada; University of Antioquia, School of Medicine, Medellín, Colombia; Pediatric Hospital Bambino Gesù, Vatican City, Italy; Department of Pediatrics, Nippon Medical School, Tokyo, Japan; Tecnologico de Monterrey School of Medicine, Monterrey, Mexico; Department of Pediatrics, Samsung Medical Center, Sungkyunkwan University School of Medicine, Seoul, Korea; Department of Pediatrics, College of Medicine and Health Sciences, United Arab Emirates University, Al-Ain, United Arab Emirates; Faculty of Medicine, University of Toronto, Toronto, ON Canada; Charité Klinik für Pädiatrie, Berlin, Germany; Department of Pediatrics, University of North Carolina, Chapel Hill, NC USA; University of Genoa, IRCCS AOU San Martino-IST, Genoa, Italy; Department of Allergy, Clinical Research Center for Allergology and Rheumatology, Sagamihara National Hospital, Kanagawa, Japan; Department of Pediatrics and Child Health, Aga Khan University Hospital, Nairobi, Kenya; Department of Paediatrics, Yong Loo Lin School of Medicine, National University of Singapore, Singapore, Republic of Singapore; Department of Primary Child Care, Children’s Hospital, Chongqing Medical University, Chongqing, China; Department of Immunology, Perth Children’s Hospital, Telethon KIDS Institute, School of Paediatrics and Child Health, University of Western Australia, Crawley, Australia; Department of Family Medicine, McMaster University, Hamilton, ON Canada; Allergy-Immunology Division, Children’s Mercy Hospital & University of Missouri – Kansas City School of Medicine, Kansas City, MO USA; Jaffe Food Allergy Institute, Icahn School of Medicine at Mount Sinai, New York, NY USA; Food Allergy Research & Education (FARE), McLean, VA USA; Department of Child and Maternal Medicine, University of Milan Medical School at the Melloni Hospital, Milan, Italy; Allergology Department, Hospital Infantil Universitario Niño Jesus, Madrid, Spain; Department of Medicine, McMaster University, Hamilton, ON Canada

**Keywords:** Allergic Diseases, Prevention, Vitamin D, Practice guidelines, GRADE

## Abstract

**Background:**

The prevalence of allergic diseases is approximately 10 % in infants whose parents and siblings do not have allergic diseases and 20**–**30 % in those with an allergic first-degree relative. Vitamin D is involved in the regulation of the immune system and it may play a role in the development, severity and course of asthma and other allergic diseases.

**Objective:**

The World Allergy Organization (WAO) convened a guideline panel to develop evidence-based recommendations addressing the use of vitamin D in primary prevention of allergic diseases.

**Methods:**

Our WAO guideline panel identified the most relevant clinical questions and performed a systematic review of randomized controlled trials and non-randomized studies (NRS), specifically cohort and case-control studies, of vitamin D supplementation for the prevention of allergic diseases. We also reviewed the evidence about values and preferences, and resource requirements (up to January 2015, with an update on January 30, 2016). We followed the Grading of Recommendations Assessment, Development and Evaluation (GRADE) approach to develop recommendations.

**Results:**

Having reviewed the currently available evidence, the WAO guideline panel found no support for the hypothesis that vitamin D supplementation reduces the risk of developing allergic diseases in children. The WAO guideline panel suggest not using vitamin D in pregnant women, breastfeeding mothers, or healthy term infants as a means of preventing the development of allergic diseases. This recommendation does not apply to those mothers and infants who have other indications for prophylactic or therapeutic use of vitamin D. The panel’s recommendations are conditional and supported by very low certainty evidence.

**Conclusions:**

WAO recommendations about vitamin D supplementation for the prevention of allergic diseases support parents, clinicians and other health care professionals in their decisions whether or not to use vitamin D in preventing allergic diseases in healthy, term infants.

**Electronic supplementary material:**

The online version of this article (doi:10.1186/s40413-016-0108-1) contains supplementary material, which is available to authorized users.

## Executive summary

Approximately 10 % of children without an allergic parent or sibling, and 20 to 30 % of those with allergies in their first-degree relatives experience allergic diseases in infancy. As demonstrated in cell culture and animal model experiments, the active form of vitamin D, calcitriol, is a modulator of both adaptive and innate immune responses [1]. Nevertheless, its role is complex and not completely understood. Additional studies relating to vitamin D’s immunomodulating function suggest it may impact the development of sensitization and allergy [2] and therefore, if administered in sufficient doses, may provide health benefits to humans by reducing a predisposition to allergic diseases.

### Methodology

The WAO-McMaster University guideline panel included allergists, paediatricians, primary care physicians, researchers in allergic diseases, and methodologists. We used the GRADEpro (www.gradepro.org) software to develop recommendations in this document following the GIN-McMaster Guideline Development Checklist (https://cebgrade.mcmaster.ca/guidecheck.html) and the Grading of Recommendations, Assessment, Development and Evaluation (GRADE) approach as applied to other WAO guidelines [3–10]. Potential conflicts of interests were managed as suggested by the World Health Organization (WHO).

The guideline panel developed and graded the recommendations, and assessed the certainty of the supporting evidence (also called confidence in the estimates of effects or quality of evidence). The certainty of the evidence is categorized as high, moderate, low or very low based on consideration of risk of bias, directness of evidence, consistency and precision of the estimates, and other considerations. Low and very low certainty evidence indicates that the estimated effects of interventions are very uncertain and any further research is very likely to influence current recommendations.

### Interpretation of strong and conditional recommendations

The strength of recommendations is expressed as either strong (guideline panel recommends**…**) or conditional (guideline panel suggests**…**) and has the following interpretation:

### Strong recommendation

- *For patients:* most individuals in this situation would want the recommended course of action, and only a small proportion would not.
- *For clinicians:* most individuals should receive the intervention. Adherence to this recommendation according to the guideline could be used as a quality criterion or performance indicator. Formal decision aids are not likely to be needed to help individuals make decisions consistent with their values and preferences.
- *For policy makers:* the recommendation can be adopted as policy in most situations.

### Conditional recommendation

- *For patients:* the majority of individuals in this situation would want the suggested course of action, but many would not.
- *For clinicians:* recognize that different choices will be appropriate for individual patients and that you must help each patient arrive at a management decision consistent with his or her values and preferences. Decision aids may be useful in helping individuals to make decisions consistent with their values and preferences.
- *For policy makers:* policy-making will require substantial debate and involvement of various stakeholders.

### How to use these guidelines

The GLAD-P guidelines about the use of vitamin D provide the basis for rational, informed decisions for clinicians, parents and other decision makers. Clinicians, patients, third-party payers, institutional review committees, other stakeholders, or the courts should not view these recommendations as dictates. No recommendation can take into account all of the often-compelling unique individual circumstances but provides guidance for typical patients. Thus, no one charged with evaluating health care professionals´ actions should apply the recommendations from these guidelines by rote or in a blanket fashion.

Note: statements regarding the underlying values and preferences as well as qualifying remarks accompanying each recommendation are integral to the recommendations and serve to facilitate more accurate interpretation; they should never be omitted when quoting recommendations from these guidelines.

## Recommendations

### Question 1: Should vitamin D be used in pregnant women?

#### Recommendation

The WAO guideline panel suggests that clinicians, parents and other decision makers do *not* use vitamin D supplementation in pregnant women with the intention of preventing the development of allergic diseases in their children (conditional recommendation, very low certainty of evidence).

#### Values and preferences

The recommendation not to use vitamin D in pregnant women with the intention of preventing the development of allergic diseases in their children places a relatively higher value on avoiding additional cost and burden and a relatively lower value on very uncertain, if any, effect on prevention of allergic diseases.

#### Explanations and other considerations

Evidence does *not* support vitamin D supplementation in pregnant women prevents the development of allergic diseases in their children. This recommendation does not apply to those pregnant women who have other indications for prophylactic or therapeutic use of vitamin D.

### Question 2: Should vitamin D be used in breastfeeding women?

#### Recommendation

The WAO guideline panel suggests that clinicians, parents and other decision makers do *not* use vitamin D supplementation in breastfeeding mothers with the intention of preventing allergic diseases their children (conditional recommendation, very low certainty evidence).

#### Values and preferences

The recommendation to not use vitamin D in breastfeeding mothers with the intention of preventing the development of allergic diseases in their children places a relatively higher value on avoiding additional cost and burden and a relatively lower value on very uncertain, if any, effect on prevention of allergic diseases.

#### Explanations and other considerations

Evidence does *not* support vitamin D supplementation in breastfeeding mothers prevents the development of allergic diseases in infants. This recommendation does not apply to those breastfeeding women who have other indications for prophylactic or therapeutic vitamin D supplementation. This recommendation does not address vitamin D supplementation for secondary prevention of allergic diseases.

### Question 3: Should vitamin D be used in infants?

#### Recommendation

The WAO guideline panel suggests that clinicians, parents and other decision makers do *not* use vitamin D supplementation in infants with the intention of preventing the development of allergic diseases (conditional recommendation, very low certainty evidence).

#### Values and preferences

The recommendation to *not* use vitamin D in healthy term infants with the intention of preventing the development of allergic diseases places a relatively higher value on avoiding additional cost and burden and a relatively lower value on very uncertain, if any, effect on prevention of allergic diseases.

#### Explanations and other considerations

Evidence does *not* support vitamin D supplementation in infants prevents the development of allergic diseases. This recommendation does not apply to infants who have other indications for prophylactic or therapeutic vitamin D supplementation. This recommendation does not address vitamin D supplementation for secondary prevention of allergic diseases.

## Scope and purpose

The purpose of this document is to evaluate the current evidence and provide guidance on the use of vitamin D for the primary prevention of allergic diseases focusing on asthma and/or wheezing, allergic rhinitis, atopic eczema (dermatitis), and food allergy. The target audiences for these guidelines are general practitioners, pediatricians, specialists in allergic disease and immunology, respiratory medicine specialists, obstetrician**/**gynaecologists, and dermatologists managing pregnant women and infants at risk of developing allergic diseases. General internists, and other health care professionals and policy makers may also benefit from these guidelines. Policy makers interested in these guidelines include those involved in developing local, national or international policies that have the goal of reducing the incidence of allergic diseases, and limiting the direct and indirect costs of allergic diseases [11]. This document may also serve as the basis for development and implementation of locally adapted guidelines.

## Introduction

Allergic diseases represent a spectrum of health conditions with a large worldwide burden. In infants, its incidence is highly influenced by the allergic status of their parents: approximately 10 % in infants without an allergic parent or sibling, versus approximately 20 to 30 % in those with an atopic history in their first-degree relatives [12].

A growing body of literature has addressed associations between vitamin D concentrations and various conditions. Observational studies and randomized trials have addressed the possible effectiveness of vitamin D supplementation in the prevention or treatment of a variety of disorders and adverse health outcomes. Emerging evidence indicates that vitamin D may play a role in the immune system. In particular, the active form of vitamin D, calcitriol, has been shown to modulate immune functioning in cell culture and animal models [13]. Nevertheless, understanding of the complex role of vitamin D in immune function remains limited [14].

The Guidelines for Atopic Disease Prevention (GLAD-P) is a joint effort of the World Allergy Organization (WAO) and the Department of Clinical Epidemiology & Biostatistics at McMaster University to evaluate the current evidence addressing the preventive effect of probiotics, prebiotics, and vitamin D on allergic diseases and related patient-important outcomes. This document provides recommendations on the rationale for use of vitamin D.

We use the following definitions throughout the document:

- Vitamin D supplement: any formulation of vitamin D, either alone or in multi-vitamin products, including products available by prescription or over the counter, and food supplements available from pharmacies and retail outlets [15].
- Family history influencing risk for allergic diseases in a child: biological parent or sibling with existing or history of allergic rhinitis, asthma, eczema, or food allergy [16].

## Methods

### Panel composition and meetings

We followed the procedures and methodology using the GIN-McMaster Guideline Development Checklist, the GRADE approach and methods we had previously applied to WAO guidelines [3–9]. Under the auspices of the organizations, we assembled a team of experts including allergists, pediatricians, and family physicians and representatives of the general public. The guideline panel included methodologists who helped to prepare systematic reviews and evidence summaries.

A face-to-face meeting was held in January 2015 coinciding with the WAO Symposium in Rome, Italy. During the meeting the guideline panel discussed specific questions in the context of existing research evidence to make recommendations.

### Disclosure of potential conflicts of interest

Guideline panel members disclosed all potential conflicts of interest according to the World Health Organization policies. The chairs (AF, RP and HJS) reviewed and resolved all potential conflicts of interest of panel members (see Additional file 1 for the list of declared conflicts of interest for all panel members). During all deliberations, panel members with potential conflicts of interest abstained from decisions about recommendations related to their potential conflict of interest.

WAO provided meeting facilities during the Symposium and financial support to perform systematic reviews. The views and interests of the WAO as well as any commercial entity that provided external funding to WAO had no influence on the final recommendations.

### Formulating specific clinical questions and determining outcomes of interest

We used the electronic tools: GRADEpro Guideline Development Tool (www.gradepro.org) [17] and SurveyMonkey (https://www.surveymonkey.com/) to brainstorm and subsequently prioritize questions related to the use of vitamin D for the prevention of allergic diseases.

The following questions were prioritized and addressed in this document:

1. Should vitamin D versus no vitamin D be used in pregnant women?
2. Should vitamin D versus no vitamin D be used in women who are breastfeeding?
3. Should vitamin D versus no vitamin D be used in infants?

The guideline selected outcomes of interest for each question, following the approach suggested by the GRADE Working Group [3]. All outcomes were identified a priori and the panel explicitly rated their relative importance for decision-making. Ranking outcomes by their relative importance can help focus attention on the outcomes that are considered most important and help to resolve or clarify potential disagreements.

### Evidence review and development of clinical recommendations

Evidence summaries for each question were prepared by the methodologists (JJYN, CCG, JB and HJS) using GRADEpro GDT (www.gradepro.org). All guideline panel members reviewed the summaries of evidence and made corrections when appropriate. We based the evidence summaries on a systematic review of the literature performed specifically for these guidelines. (Reference. in preparation). An updated search strategy (presented in the online Additional file 2) was performed on January 30, 2016 that provided two additional studies. We followed the methods of the Cochrane Collaboration (handbook.cochrane.org) and assessed the risk of bias at the outcome level using the Cochrane Collaboration**’**s risk of bias tool [18], and version 1.0 of the Cochrane Risk of Bias Assessment Tool for Non-Randomized Studies of Interventions, now called ROBINS-I [19], for RCTs and NRSs, respectively. Subsequently, we assessed the certainty of the body of evidence (confidence in the estimated effects) for each of the outcomes of interest using the GRADE approach based on the consideration of risk of bias, directness of evidence, consistency and precision of the estimates, and other factors such as publication bias.

We searched for evidence about values and preferences and cost of vitamin D supplementation. We prepared the evidence-to decision frameworks based on the estimates of the health effects, values and preferences, and resource use.

During the meeting, the guideline panel developed recommendations based on the evidence summaries and the evidence-to-decision frameworks. For each recommendation, the guideline panel considered and agreed on the following: the certainty of the evidence, the balance of desirable and undesirable consequences of compared management options, the feasibility, acceptability and impact on health inequities for each recommendation, as well as the assessment of the values and preferences associated with the decision. The guideline panel also explicitly took into account the possible extent of resource use associated with alternative management options.

Recommendations and their strength were developed through consensus and no recommendation required voting. The panel agreed on the final wording of recommendations and remarks with further qualifications for each recommendation.

We labelled the recommendations as either **“**strong**”** or **“**conditional**”** according to the GRADE approach. We used the words **“**the panel members recommend**”** for strong recommendations and **“**suggest**”** for conditional recommendations. Table 1 provides suggested interpretation of strong and conditional recommendations.

**Table 1:** Interpretation of strong and conditional recommendations

Implications for:	Strong recommendation	Conditional recommendation
Patients	Most individuals in this situation would want the recommended course of action, and only a small proportion would not.	The majority of individuals in this situation would want the suggested course of action, but many would not.
Clinicians	Most individuals should receive the intervention. Adherence to this recommendation according to the guideline could be used as a quality criterion or performance indicator. Formal decision aids are not likely to be needed to help individuals make decisions consistent with their values and preferences.	Recognize that different choices will be appropriate for individual patients and that you must help each patient arrive at a management decision consistent with his or her values and preferences. Decision aids may be useful in helping individuals to make decisions consistent with their values and preferences.
Policy makers	The recommendation can be adopted as policy in most situations.	Policymaking will require substantial debate and involvement of various stakeholders.

## Document review

Each member of the guideline panel reviewed the final draft document and approved the document, which was then submitted to the WAO for peer review. The document was revised to incorporate the pertinent comments suggested by the external reviewers.

### How to use these guidelines

The WAO-McMaster University GLAD-P guidelines about the use of vitamin D in the primary prevention of allergic diseases in children are not intended to impose a standard of care. They provide the basis for rational decisions. Clinicians, patients, third-party payers, institutional review committees, other stakeholders, or the courts should never view these recommendations as dictates. No recommendation can take into account all of the often-compelling unique circumstances of each individual patient. Therefore, no one charged with evaluating health care professionals’ actions should apply the recommendations from these guidelines as rote or in a blanket fashion.

Statements regarding the underlying values and preferences as well as qualifying remarks accompanying each recommendation are integral parts and serve to facilitate more accurate interpretation. They should never be omitted when quoting recommendations from these guidelines.

## Recommendations

### Question 1. Should vitamin D versus no vitamin D be used in pregnant women?

#### Summary of the evidence

We found no systematic review addressing this question. We found seven publications [reporting six randomized control trials (RCTs)] that investigated the effects of vitamin D supplementation in pregnant women. Only one RCT [20] measured the risk of developing allergic diseases in children, and the remaining trials [21–27] reported nutritional status, adverse effects, and development of rickets. No study reported quality of life and development of a composite of “any allergy”. We also found two observational studies, one case-control and one cohort study, which reported the effect of vitamin D supplementation in pregnant women on the development of food allergy and wheezing in their infants respectively [28, 29].

The randomized trial failed to detect an effect of vitamin D supplementation on the risk of developing allergic diseases in children: atopic dermatitis (RR 0.96, 95 % CI 0.57 to 1.61), allergic rhinitis (RR 0.76, 95 % CI 0.31 to 1.85), asthma and/or wheezing (RR 1.12, 95 % CI 0.50 to 2.54), and food allergy (RR 1.92, 95 % CI 0.57 to 6.50). Vitamin D supplementation in pregnant women was not associated with a lower birth weight in infants compared to no vitamin D (mean difference 52.78 g more, 95 % CI -64.34 to 169.90). Adverse effects as measured by infants being born small for gestational age, gestational age, symptomatic hypocalcaemia, as well as severe adverse effects in a newborn and in the mother were very infrequent and any estimates are very imprecise. There was no difference between the groups.

The findings of the case-control study were consistent with the findings of the randomized trial for food allergy (OR 1.50, 95 % CI 0.78 to 2.88). In the cohort study, vitamin D was associated with low risk of developing wheezing (OR 0.65, 95 % CI 0.46 to 0.93). The overall certainty of the body of evidence was very low owing to the risk of bias and imprecision. Online Additional file 3 presents the characteristics of all included studies for all questions. 

#### Benefits

Thus far, there is no direct evidence from clinical studies suggesting that vitamin D supplementation during pregnancy reduces the risk of developing allergic diseases in children (see evidence profile for question 1 in the Additional file 4).

#### Harms and burden

Adverse effects in pregnant women were well defined in all studies. Abnormal glucose challenge test was reported as an adverse effect in one study [22]. Other adverse effects included nausea and vomiting [26]. Maternal serious adverse effects included gastroenteritis, preterm delivery with premature rupture of membranes, injury, and pre-eclampsia**.** For pregnant women with adverse effects and serious adverse effects, there was no difference among those who received vitamin D and those who did not receive vitamin D (RR 1.46, 95 % CI 0.31 to 6.78; RR 1.55, 95 % CI 0.65 to 3.69 respectively). The certainty in the estimate of the risk of adverse events in pregnant mothers ranged between low to very low due to the development of few events in both arms.

Adverse effects in infants were well documented and included low birth weight (assessed in grams), gestational age (preterm labour), small for gestational age (assessed as weight lower than 10th centile or less than 2.5 kg) (RR 0.67, 95 % CI 0.37 to 1.19), hypocalcaemia (RR 0.10, 95 % CI 0.01 to 1.82), any and serious adverse effects (RR 0.79, 95 % CI 0.33 to 1.90). No adverse effects related to the administration of vitamin D in infants were reported in two studies [20, 21]. Serious adverse effects included hypoxic-ischemic encephalopathy, meconium aspiration syndrome, jaundice, neonatal seizures, sepsis, sub-acute intestinal obstruction, pneumonia, intrauterine, and neonatal death. In total, three intrauterine and four neonatal deaths (two and one deaths in the vitamin D group respectively) were reported. Reasons for intrauterine deaths were not specified. A preterm infant with very low birth weight, died within 5 min of birth at home (placebo group). The reasons for other neonatal deaths were sepsis and multi-organ failure, severe hypoxic-ischemic encephalopathy and respiratory failure, cardio-respiratory failure of unknown etiology. Adverse effects were not different among infants who received vitamin D and those who did not receive vitamin D. The certainty of the evidence of adverse effects in infants ranged between moderate (gestational age, birth weight), low (weight at 1 year, any, and serious adverse events), and very low (small for gestational age, symptomatic hypocalcaemia). The certainty in the estimates was assessed as moderate and low confidence due to imprecision in the estimates. Very low certainty in the estimates was downgraded due to imprecision and risk of bias.

#### Decision criteria and considerations

The values and preferences of women regarding the use of vitamin D during pregnancy are likely dependent on cultural and socioeconomic background. We explicitly considered the required resources. Prices of vitamin D are likely to vary depending on the factors such as country, region or formulation. The literature review did not identify any cost-effectiveness analysis related to the preventive use of vitamin D for allergic diseases.

#### Conclusions and research needs

The guideline panel determined that it is unlikely that there is a net benefit from using vitamin D in pregnant women who have no specific indications for prophylactic or therapeutic vitamin D supplementation and the only purpose would be to primary prevention of allergic diseases in their children. We found no impact of vitamin D on the development of allergic diseases. There is a need for a rigorously designed and executed randomized trial of vitamin D supplementation in pregnant women that would properly measure and report patient-important outcomes, including development of allergic diseases, quality of life, and adverse effects. Long-term follow-up of such studies to evaluate long-term effects is also needed.

#### What others are saying

We found no guidelines that made specific recommendations about the use of vitamin D in pregnancy with the intention of preventing allergic diseases in children. The World Health Organization (WHO) states “Vitamin D supplementation is not recommended during pregnancy to prevent the development of pre-eclampsia and its complications (strong recommendation). In addition, due to the limited evidence currently available to directly assess the benefits and harms of the use of vitamin D supplementation alone in pregnancy for improving maternal and infant health outcomes, the use of this intervention during pregnancy as part of routine antenatal care is also not recommended (conditional recommendation) [30]”.

The National Institute for Health and Care Excellence (NICE) recommends increasing the access to vitamin D supplements in high risk groups that have a low vitamin D status such as pregnant and breastfeeding women, particularly teenagers and young women [15]. The American Academy of Pediatrics (AAP) [31] recommends, on an individual basis, pregnant women should receive adequate amounts of vitamin D3 to ensure that her 25-OH-D levels are sufficiently high (>80 nmol/L). Similarly, the Endocrine Society Clinical Practice Guideline [32] states that pregnant women require adequate supplementation of vitamin D so that vitamin D levels are not insufficient.

The European Society for Paediatric Gastroenterology, Hepatology, and Nutrition Committee on Nutrition [33], the European Academy of Allergy and Clinical Immunology [34], Food Allergy and Anaphylaxis Guidelines [35] and the Guidelines from the US National Institute of Allergy and Infectious Diseases [16], make no specific recommendations regarding the use of vitamin D in pregnant women.

#### Recommendation 1

The WAO guideline panel suggests that clinicians, parents and other decision makers do *not* use vitamin D supplementation in pregnant women with an intention to prevent development of allergic diseases in their children (conditional recommendation, very low certainty of evidence).

#### Values and preferences

The recommendation not to use vitamin D in pregnant women with the intention of preventing the development of allergic diseases in their children places a relatively higher value on avoiding additional cost and burden and a relatively lower value on very uncertain, if any, effect on prevention of allergic diseases.

#### Explanations and other considerations

Evidence does *not* support vitamin D supplementation in pregnant women prevents the development of allergic diseases in their children. This recommendation does not apply to those pregnant women who have other indications for prophylactic or therapeutic use of vitamin D. See the evidence to recommendation table for question 1 in online Additional file 5.

### Question 2. Should vitamin D versus no vitamin D be used in breastfeeding mothers?

#### Summary of the evidence

We found no systematic review or randomized trial of vitamin D supplementation in breastfeeding mothers that reported on the development of allergic diseases in children. We identified one case-control study which addressed this question and found no effect of vitamin D supplementation in breastfeeding mothers on the risk of developing asthma and/or wheezing in children: (OR 1.09, 95 % CI 0.84 to 1.40) [36].

One RCT [37] reported no cases of rickets among 60 children of breastfeeding mothers that either used or didn’t use vitamin D supplements. The time of follow-up was 6 weeks, which was considered too short to develop this outcome. No experimental or observational study with an independent control group reported other outcomes of interest. Adverse effects were not reported in the included studies. The overall certainty of the body of evidence was very low.

#### Benefits

There is no evidence from clinical studies suggesting an effect of vitamin D supplementation in breastfeeding mothers on the risk of developing allergic diseases in children (see evidence profile for question 2 in the Additional file 4).

#### Harms and burden

Any estimate of potential adverse effects is very uncertain due to the unavailability of reports of adverse effects.

#### Decision criteria and considerations

The panel agreed that the considerations of values and preferences, resource implications, and equity are likely similar to those in pregnant women.

#### Conclusions and research needs

The guideline panel determined that it is not possible to determine whether there is any benefit from using vitamin D supplementation in breastfeeding women who have no specific indications for prophylactic or therapeutic vitamin D supplementation for the sole purpose of reducing the risk of developing allergic diseases in otherwise healthy term children. Given the paucity of information, it is not possible to exclude an appreciable benefit, no effect or appreciable harm.

There is a need for a rigorously designed and well-executed randomized trial of vitamin D in breastfeeding women that would properly measure and report patient-important outcomes, including quality of life and adverse effects. Long-term follow-up of such studies to evaluate long-term effects is also needed.

#### What others are saying

We found no guidelines that made specific recommendations about the use of vitamin D in breastfeeding mothers with the intention of preventing allergic diseases in children. The AAP [31] does not recommend supplementing breastfeeding mothers with high doses of vitamin D in order to increase the 25-OH-D concentrations in their breastfed infants. However, they recommend, “a supplement of 400 IU/day of vitamin D should begin within the first few days of life and continue throughout childhood”. NICE [15] recommends increasing the access to vitamin D supplements in high risk groups of low vitamin D status such as pregnant and breastfeeding women, particularly teenagers and young women. ESPGHAN [33], considering the prevalence of vitamin D deficiency in pregnant mothers, recommends that a higher vitamin D supply in preterm infants could be necessary in order to correct the foetal low plasma level. This statement holds for both premature infants fed mother’s milk and those fed formula milk.

WHO [30], the Endocrine Society Clinical Practice Guideline [32], the European Academy of Allergy and Clinical Immunology [34] Food Allergy and Anaphylaxis Guidelines [35] and the Guidelines from the US National Institute of Allergy and Infectious Diseases [16], make no specific recommendations about the use of vitamin D in breastfeeding women.

#### Recommendation 2

The WAO guideline panel suggests that clinicians, parents and other decision makers do *not* use vitamin D supplementation in breastfeeding mothers with the intention of preventing allergic diseases in their children (conditional recommendation, very low certainty evidence).

#### Values and preferences

This recommendation not to use vitamin D in breastfeeding mothers with an intention of preventing the development of allergic diseases in their children places a relatively higher value on avoiding additional cost and burden and a relatively lower value on very uncertain, if any, effect on prevention of allergic diseases.

#### Explanations and other considerations

Evidence does not support that vitamin D supplementation in breastfeeding mothers prevents the development of allergic diseases in infants. This recommendation does not apply to those breastfeeding women who have other indications for prophylactic or therapeutic vitamin D supplementation. This recommendation does not address vitamin D supplementation for secondary prevention of allergic diseases. See the evidence to recommendation table for question 2 in online Additional file 5.

### Question 3. Should vitamin D vs. no vitamin D be used in healthy infants?

#### Summary of the evidence

We found no systematic review addressing this question. We found 5 randomized trials that investigated vitamin D supplementation in infants but none reported allergy outcomes [38–42]. One RCT [42] could not be meta-analyzed because it reported mean values without measures of dispersion. We identified three NRS. Two cohort studies reported the risk of developing allergic rhinitis and asthma [43], and wheezing/asthma [29]. The case control study measured development of food allergy [28]. No study measured quality of life and development of eczema or a composite of “any allergy”.

In the first cohort study [43], regular vitamin D supplementation during the first year of life increased the risk of developing allergic rhinitis (RR 1.95, 95 % CI 0.69 to 5.54) but there were only three events among 20 children in the control group, which makes these results very fragile and very imprecise. Study authors combined the group that never received vitamin D with a group that used it irregularly to reduce fragility of the results – if those using vitamin D regularly were compared with those who either did not use it, or used it irregularly, the RR would be 1.31 (95 % CI 1.15 to 1.49). Irrespective of the choice of control group, there is a high risk of bias in these estimates, because results were not adjusted for confounding factors despite the fact that “cohort members with a family history of asthma were less likely to receive supplementation according to recommendations (…) and many of the same characteristics that were predictive of worse compliance were associated with reduced risk of allergies” [43]. In the same study, the risk of developing asthma and/or wheezing was estimated too imprecisely to make any conclusion about the effect (RR 3.07, 95 % CI 0.19 to 50.88). If those using vitamin D regularly were compared with those who either did not use it or used it irregularly, the RR would be 1.36 (95 % CI 1.00 to 1.85). The certainty of the evidence for these two outcomes was very low.

The second cohort study [29] did not show an effect in the primary prevention of asthma/wheezing in infants if they were exposed to vitamin D supplementation during their chilhood (OR 1.00, 95 % CI 0.81 to 1.23).

In the case-control study the risk of developing food allergy during the first year of life was reduced in infants who received vitamin D compared to those who did not (OR 0.49, 95 % CI 0.27 to 0.88) [28]. However, the confidence in this estimate is also very low owing to indirectness of the evidence and risk of bias.

Four RCTs reported the estimation of developing rickets. None of the infants in these four studies developed rickets. One RCT reported weight at one year of age without significant differences between those infants who received vitamin D and those who did not receive vitamin D. Information about weight was available only graphically. The certainty of evidence ranged between low to very low owing to imprecision, risk of bias, and indirectness.

Adverse effects were reported in three RCTs. These adverse effects included inter-current acute diseases, and urinary tract infection. As a serious adverse effect, one study reported a sudden infant death syndrome, which was not related to the trial. Adverse effects and serious adverse effects were not different among infants who received vitamin D and those who did not receive vitamin D (RR 0.85, 95 % CI 0.23 to 3.14; RR 0.17, 95 % CI 0.01 to 3.70 respectively). The certainty of the evidence was very low due to serious risk of bias and imprecision.

No RCT or NRS addressed the efficacy or association of primary prevention of allergic diseases in children (over 2 years old) after vitamin D supplementation.

#### Benefits

There is a probable reduction in the risk of developing food allergy in infants during the first year of life (RR: 0.49, 95 % CI: 0.27 to 0.88) with vitamin D supplementation. There is no evidence supporting a reduction in the risk of developing any other allergic disease in infants (see evidence profile for question 3 in the Additional file 4).

#### Harms and burden

Regular vitamin D supplementation during the first year of life increased the risk of developing allergic rhinitis (RR 1.95, 95 % CI 0.69 to 5.54), and asthma and/or wheezing (RR 3.07, 95 % CI 0.19 to 50.88; OR 1.00, 95 % CI 0.81 to 1.23) but the certainty of the evidence is very low.

#### Decision criteria and considerations

If vitamin D is used in infants, it is not clear when it should be started and how long it should be used and there is an uncertainty about the dosage.

The previous considerations concern otherwise healthy infants in whom vitamin D would be used for primary prevention of allergic diseases. They do not concern using vitamin D for specific indications, e.g., preterm infants, especially with birth weight <1800 to 2000 g [35].

#### Conclusions and research needs

The guideline panel determined that net benefit from using vitamin D in infants is uncertain. There is a need for rigorously designed and well executed randomized trials of vitamin D in infants that would measure and adequately report patient-important outcomes, including adverse effects.

#### What others are saying

We found no guidelines that made specific recommendations about the use of vitamin D in infants with the intention of preventing development of allergic diseases. NICE [15] recommends increasing the access to vitamin D supplements in high risk groups of low vitamin D status such as infants and children aged under 5. AAP [31] recommends that “infants who receive a mixture of human milk and formula also should get a vitamin D supplement of 400 IU/day to ensure an adequate intake. Additionally, “any infant who receives <1 L or 1 qt of formula per day needs an alternative way to get 400 IU/day of vitamin D, such as through vitamin supplements”. The Endocrine Society Clinical Practice Guideline [32] suggests that infants and children age 0-1 year require an intake of vitamin D of at least 400 IU/d, and children 1 year and older, at least 600 IU/d to maximize bone health. ESPGHAN [39] recommends that, considering the prevalence of vitamin D deficiency in pregnant mothers, higher vitamin D supply in preterm infants could be necessary to rapidly correct the foetal low plasma level. A vitamin D intake of 800 to 1000 IU/day (and not per kilogram) during the first months of life is recommended. WHO [30] and the European Academy of Allergy and Clinical Immunology Food Allergy and Anaphylaxis Guideline [35] make no specific recommendations about the use of vitamin D in infants.

#### Recommendation 3

The WAO guideline panel suggests that clinicians, parents and other decision makers do *not* use vitamin D supplementation in infants with an intention to prevent development of allergic diseases (conditional recommendation, very low certainty evidence).

#### Values and preferences

This recommendation to not use vitamin D in healthy term infants with the intention of preventing the development of allergic diseases places a relatively higher value on avoiding additional cost and burden and a relatively lower value on very uncertain, if any, effect on prevention of allergic diseases.

#### Explanations and other considerations

Evidence does *not* support that vitamin D supplementation in infants prevents the development of allergic diseases. This recommendation does not apply to infants who have other indication for prophylactic or therapeutic vitamin D supplementation. This recommendation does not address vitamin D supplementation for secondary prevention of allergic diseases. See the evidence to recommendation table for question 3 in online Additional file 5.

#### Priorities for revision of the guidelines

##### Plans for updating these guidelines

To remain useful, guidelines need to be updated regularly as new information accumulates. A revision of this document will be needed, because there was limited evidence for the three clinical questions.

This document will be updated when major new research is published. As it was stated before, this guideline was focused on primary prevention. However we will look at secondary prevention in the next update of this guideline. The need for updates will be determined no later than in 2019.

##### Updating or adapting recommendations locally

The methods used to develop these guidelines are transparent. The recommendations have been developed to be as specific and detailed as possible without losing sight of the desirability of simplicity. Since GLAD-P guidelines are meant as international guidelines, the panel encourages feedback on all aspects including their applicability in individual countries. The panel will consider this feedback when revising the document.

Adaptation of these guidelines will be necessary in many circumstances. Depending on when such a process takes place, the following steps should be taken:

- Appointing a guideline committee comprised of clinicians and methodologists.
- Determining the scope of the localized guidelines.
- Defining the clinical questions to be addressed.
- Updating the evidence profiles and evidence-to decision frameworks, if necessary.
- Reviewing the recommendations in the GLAD-P guidelines (the recommendations may need to be modified at a local level, depending on the local values and preferences, availability of medications, costs, etc.).
- Disseminating the guidelines, with a clear **“**use by**”** date.
- Developing a method to obtain feedback and plans for review and update.

#### Priorities for research

During the guideline development process we identified a need for more data on specific topics. This results in the following recommendations for research. We summarize these gaps in the evidence as research recommendations, to assist those in a position to provide such information by the design and execution of specific research projects.

#### Specific research needs to be addressed:

- Development of clinical prediction guides for evaluating the risk of allergic diseases in children (the family history predicts only about 30 % of the population risk).
- Evaluation of effects of using vitamin D in breastfeeding mothers specifically in that period (as opposed to intervention administered during pregnancy and to children).
- Evaluation of the effects of different ways of administering vitamin D, e.g., as milk or dairy supplements, stand-alone supplements, etc.
- Performance of rigorously designed, adequately powered, and well executed randomized trials of vitamin D in infants who did not receive vitamin D prenatally and/or during breastfeeding; studies should include infants considered to be at high and low/average risk for allergic diseases and should properly report patient-important outcomes, including adverse effects. The estimated optimal information size for this question is from approximately 2500 participants (for eczema) to 27,000 participants (for food allergy). However, for the evaluation of adverse effects, a large compilation of RCTs as well as observational studies might be necessary with thousands of observations.
- Evaluation addressing which of the 3 populations (pregnant women, breastfeeding mothers, and infants) should receive vitamin D **–** whether there is a larger benefit with supplementation in one or a combination of these populations and, if so, which populations to target.

## Additional files

Additional file 1: Declaration of potential conflicts of interest (within last 4 years). (DOCX 500 kb) Additional file 2: Search strategies. (DOC 152 kb) Additional file 3: Characteristics of included studies. (DOC 124 kb) Additional file 4: Evidence profiles. (DOCX 79 kb) Additional file 5: Evidence to Decision table. (DOC 196 kb)
