# Supplementary material for: World Allergy Organization-McMaster University Guidelines for Allergic Disease Prevention (GLAD-P): Vitamin D
Source: World Allergy Organ J. 2016 May 17;9:17. doi: 10.1186/s40413-016-0108-1 (PMC4869275; doi:10.1186/s40413-016-0108-1)
Supplement: Additional file 2: — Search strategies. (DOC 152 kb) [file 40413_2016_108_MOESM2_ESM.doc]

| **Additional file 2. Search strategies** | | | |
| --- | --- | --- | --- |
| **Interface** Ovid | | | |
| **Database *Medline*** | | | |
| **Date of searching** January 30, 2016 | | | |
| **Study Types** Randomized control trials and observational studies^†^ | | | |
| **Limits** No limits | | | |
| **Database strategy** | | | |
| 1 | exp Vitamin D/ | | 46990 |
| 2 | hydroxyvitamin D.mp. | | 9536 |
| 3 | 1,25-dihydroxycholecalciferol.mp. | | 1125 |
| 4 | 24,25-dihydroxycholecalciferol.mp. | | 155 |
| 5 | 1,25-dihydroxyvitamin D3.mp. | | 4713 |
| 6 | Vitamin D.tw. | | 43639 |
| 7 | 1 or 2 or 3 or 4 or 5 or 6 | | 65198 |
| 8 | exp Child/ | | 1637039 |
| 9 | exp Infant/ | | 991473 |
| 10 | Childhood.mp. | | 194126 |
| 11 | Infant*.mp. | | 1083596 |
| 12 | Infancy.tw. | | 40952 |
| 13 | Baby.tw. | | 29822 |
| 14 | Baby's.tw. | | 1767 |
| 15 | Babies.tw. | | 30029 |
| 16 | Neonat*.mp. | | 232911 |
| 17 | Child*.mp. | | 2014319 |
| 18 | Schoolchild*.tw. | | 11251 |
| 19 | Schoolage*.tw. | | 60 |
| 20 | School age*.tw. | | 15763 |
| 21 | Kids.tw. | | 4276 |
| 22 | Kid.tw. | | 1348 |
| 23 | Toddler*.tw. | | 6943 |
| 24 | exp Pediatrics/ | | 48238 |
| 25 | Pediatric*.mp. | | 243725 |
| 26 | Paediatric*.mp. | | 45757 |
| 27 | exp Schools/ | | 93035 |
| 28 | Kindergar*.tw. | | 4754 |
| 29 | Primary school*.mp. | | 8699 |
| 30 | exp Pregnancy/ | | 778451 |
| 31 | Pregnant Women/ | | 5514 |
| 32 | Gestation.mp. | | 96310 |
| 33 | (pregnanc* or pregnant).mp. | | 844193 |
| 34 | gravidity/ or postpartum period/ or pregnancy trimesters/ | | 22478 |
| 35 | (gravidity or peripartum period or postpartum period).mp. | | 27479 |
| 36 | exp Breast Feeding/ | | 30816 |
| 37 | (breast feeding or breastfeeding).mp. | | 40802 |
| 38 | Lactation/ | | 34864 |
| 39 | lactation.mp. | | 48150 |
| 40 | Milk, Human/ | | 16202 |
| 41 | ((human or breast) adj1 milk).mp. | | 22920 |
| 42 | or/8-41 | | 3318393 |
| 43 | 7 and 42 | | 11604 |
| 44 | exp animals/ not humans.sh. | | 4177874 |
| 45 | 43 not 44 | | 10694 |
| 46 | exp Hypersensitivity/ | | 299314 |
| 47 | hypersensitiv*.mp. | | 154466 |
| 48 | allerg*.mp. | | 182301 |
| 49 | anaphyla*.mp. | | 31973 |
| 50 | asthma*.mp. | | 147962 |
| 51 | wheez*.mp. | | 10884 |
| 52 | eczema*.mp. | | 19485 |
| 53 | dermatit*.mp. | | 77572 |
| 54 | rhinit*.mp. | | 36427 |
| 55 | hyperreactiv*.mp. | | 12121 |
| 56 | (atopi* or atopy).mp. | | 38578 |
| 57 | or/46-55 | | 483374 |
| 58 | 45 and 57 | | 538 |
| ^†^Case-control and cohort studies | | | |
|  | |  | |

|  |
| --- |

|  |
| --- |

| **Interface** Ovid | | |
| --- | --- | --- |
| **Database *Embase*** | | |
| **Date of searching** January 30, 2016 | | |
| **Study Types** Randomized control trials and observational studies^†^ | | |
| **Limits** No limits | | |
| **Database strategy** | | |
| 1 | exp Vitamin D/ | 105925 |
| 2 | hydroxyvitamin D.mp. | 15624 |
| 3 | 1,25-dihydroxycholecalciferol.mp. | 1334 |
| 4 | 24,25-dihydroxycholecalciferol.mp. | 163 |
| 5 | 1,25-dihydroxyvitamin D3.mp. | 6018 |
| 6 | Vitamin D.tw. | 62288 |
| 7 | 1 or 2 or 3 or 4 or 5 or 6 | 117168 |
| 8 | exp Child/ | 2272928 |
| 9 | exp Infant/ | 926161 |
| 10 | Childhood.mp. | 330316 |
| 11 | Infant*.mp. | 803451 |
| 12 | Infancy.tw. | 51760 |
| 13 | Baby.tw. | 40153 |
| 14 | Baby's.tw. | 2470 |
| 15 | Babies.tw. | 40269 |
| 16 | Neonat*.mp. | 279324 |
| 17 | Child*.mp. | 2270276 |
| 18 | Schoolchild*.tw. | 13113 |
| 19 | Schoolage*.tw. | 267 |
| 20 | School age*.tw. | 20183 |
| 21 | Kids.tw. | 5607 |
| 22 | Kid.tw. | 1858 |
| 23 | Toddler*.tw. | 9009 |
| 24 | exp Pediatrics/ | 89907 |
| 25 | Pediatric*.mp. | 381375 |
| 26 | Paediatric*.mp. | 77246 |
| 27 | exp Schools/ | 281779 |
| 28 | Kindergar*.tw. | 5745 |
| 29 | Primary school*.mp. | 115091 |
| 30 | exp Pregnancy/ | 638165 |
| 31 | Pregnant Women/ | 431318 |
| 32 | Gestation.mp. | 127611 |
| 33 | (pregnanc* or pregnant).mp. | 828937 |
| 34 | gravidity/ or postpartum period/ or pregnancy trimesters/ | 605720 |
| 35 | (gravidity or peripartum period or postpartum period).mp. | 10771 |
| 36 | exp Breast Feeding/ | 38888 |
| 37 | (breast feeding or breastfeeding).mp. | 48741 |
| 38 | Lactation/ | 41982 |
| 39 | lactation.mp. | 52471 |
| 40 | Milk, Human/ | 20880 |
| 41 | ((human or breast) adj1 milk).mp. | 28541 |
| 42 | or/8-41 | 3862110 |
| 43 | 7 and 42 | 21988 |
| 44 | exp animals/ not humans.sh. | 21055025 |
| 45 | 43 not 44 | 2362 |
| 46 | exp Hypersensitivity/ | 514647 |
| 47 | hypersensitiv*.mp. | 157307 |
| 48 | allerg*.mp. | 304176 |
| 49 | anaphyla*.mp. | 56335 |
| 50 | asthma*.mp. | 242104 |
| 51 | wheez*.mp. | 23991 |
| 52 | eczema*.mp. | 37471 |
| 53 | dermatit*.mp. | 105183 |
| 54 | rhinit*.mp. | 49945 |
| 55 | hyperreactiv*.mp. | 17775 |
| 56 | (atopi* or atopy).mp. | 63796 |
| 57 | or/46-55 | 714556 |
| 58 | 45 and 57 | 96 |
| ^†^Case-control and cohort studies | | |

| **Database *The Cochrane Library*** | | |
| --- | --- | --- |
| **Date of searching** January 30, 2016 | | |
| **Study Types** Randomized control trials and observational studies* | | |
| **Limits** No limits | | |
| **Database strategy** | | |
| 1 | MeSH descriptor: [Vitamin D] explode all trees | 2805 |
| 2 | "vitamin D":ti,ab,kw | 4621 |
| 3 | hydroxyvitamin | 1427 |
| 4 | 1,25-dihydroxycholecalciferol | 59 |
| 5 | 1,25-dihydroxyvitamin D3 | 171 |
| 6 | 24,25-dihydroxycholecalciferol | 9 |
| 7 | #1 or #2 or #3 or #4 or #5 or #6 | 5685 |
| 8 | MeSH descriptor: [Child] explode all trees | 152 |
| 9 | MeSH descriptor: [Infant] explode all trees | 14330 |
| 10 | Infant* | 43342 |
| 11 | Infancy | 1655 |
| 12 | Baby* | 2871 |
| 13 | Babies | 3380 |
| 14 | Neonat* | 21029 |
| 15 | Preterm* | 7785 |
| 16 | Newborn* | 19707 |
| 17 | Prematur* | 15107 |
| 18 | Postmatur* | 35 |
| 19 | Child* | 106611 |
| 20 | Schoolchild* | 1062 |
| 21 | School age* | 52028 |
| 22 | Kid | 106 |
| 23 | Kids* | 503 |
| 24 | Toddler* | 948 |
| 25 | Boy* | 8480 |
| 26 | Girl* | 4732 |
| 27 | MeSH descriptor: [Minors] explode all trees | 8 |
| 28 | Minors* | 197 |
| 29 | MeSH descriptor: [Pediatrics] explode all trees | 608 |
| 30 | Paediatric* | 10981 |
| 31 | Peadiatric* | 42 |
| 32 | MeSH descriptor: [Schools] explode all trees | 1991 |
| 33 | Preschool* | 31280 |
| 34 | Kindergar* | 456 |
| 35 | Primary school* | 15081 |
| 36 | #8 or #9 or #10 or #11 or #12 or #13 or #14 or #15 or #16 or #17 or #18 or #19 or #20 or #21 or #22 or #23 or #24 or #25 or #26 or #27 or #28 or #29 or #30 or #31 or #32 or #33 or #34 or #35 | 177870 |
| 37 | MeSH descriptor: [Pregnancy] explode all trees | 6296 |
| 38 | MeSH descriptor: [Breast Feeding] explode all trees | 1498 |
| 39 | #36 or #37 or #38 | 180453 |
| 40 | #7 and #39 | 1298 |
