# Supplementary material for: World Allergy Organization-McMaster University Guidelines for Allergic Disease Prevention (GLAD-P): Vitamin D
Source: World Allergy Organ J. 2016 May 17;9:17. doi: 10.1186/s40413-016-0108-1 (PMC4869275; doi:10.1186/s40413-016-0108-1)
Supplement: Additional file 3: — Characteristics of included studies. (DOC 124 kb) [file 40413_2016_108_MOESM3_ESM.doc]

| **Additional file 3. Characteristics of included studies** | | | | | | | | |
| --- | --- | --- | --- | --- | --- | --- | --- | --- |
| **1. PREGNANT WOMEN** | | | | | | | | |
| **Study, Year** | **n** | **Population who received vitamin D** | **Type of vitamin D administered** | **Route of administration and forms** | | **Doses of vitamin D administered** | **Time of administration of vitamin D** | **Time of follow-up for outcome(s)** |
| ***Randomized control trials*** | | | | | | | | |
| Brooke, 1980 | 126* | **Pregnant women** with 28 gestation weeks | Ergocalciferol | Oral (unreported) | 1000 IU/day | | Until delivery | At birth |
| Goldring, 2013 | 180* | **Pregnant women** at 27 weeks of gestation | Vitamin D3 (ergocalciferol) | Oral (tablets) | 800 IU/day or 200000 UI (single dose)** | | 2 months (until delivery) | At 36 months |
| Hossain, 2014 | 200* | **Pregnant women** at 20 weeks of gestation | Vitamin D3 (Ergocalciferol) | Oral (liquid formulation) | 4000 IU/day | | Until delivery | At birth |
| Mallet, 1986 | 57* | **Pregnant women** at seven month | Vitamin D2 | Unreported | 1000 IU/day or 200000/IU (single dose) | | Last trimester of pregnancy | At birth |
| Roth, 2013-A | 160* | **Pregnant women** with gestational age of 26 to < 30 weeks | Vitamin D3 (cholecalciferol) | Oral (liquid formulation) | 35000 IU/week | | 3 months (last trimester of pregnancy) | At birth |
| Roth, 2013-B | 160* | **Pregnant women** with gestational age of 26 to < 30 weeks | Vitamin D3 (cholecalciferol) | Oral (liquid formulation) | 35000 IU/week | | 3 months (last trimester of pregnancy) | One year after delivery |
| Sablok, 2015 | 180* | **Pregnant women** with gestational age at 14–20 weeks | Vitamin D (cholecalciferol) | Oral | Between 60000 IU to 480000 IU | | Between second to third trimester of pregnancy | At birth |
| Yu, 2009 | 180* | **Pregnant women** with 27 gestation weeks | Vitamin D (Ergocalciferol or calciferol) | Oral | 800 UI/day or 200000 IU (single dose) | | Until delivery | At birth |
| ***Non-randomized studies*** | | | | | | | | |
| Allen, 2013 | 2252 | **Pregnant women** | Maternal use of vitamin D supplement | Unreported | | Unreported | During pregnancy | Infants with aged between 11 and 15 months |
| Anderson 2015 | 2478 | **Pregnant women** | Maternal use of vitamin D supplement | Unreported | | 400 or 1000 IU/day | During pregnancy | Infants with aged between 0 to 5 years |
|  |  |  |  |  | |  |  |  |
| **2. BREASTFEEDING** | | |  |  | |  |  |  |
| **Study, Year** | **n** | **Population who received vitamin D** | **Type of vitamin D administered** | **Route of administration and forms** | | **Doses of vitamin D administered** | **Time of administration of vitamin D** | **Time of follow-up for outcome(s)** |
| ***Randomized control trials*** | | | | | | | | |
| Rothberg, 1982 | 60* | **Breastfeeding mothers** | Vitamin D | Unreported | | 500 IU or 1000 IU/day | 6 weeks | At 6 weeks of age |
| ***Non-randomized studies*** | | | | | | | | |
| Bener, 2012 | 966 | **Breastfeeding mothers** | Vitamin D supplementation | Unreported | | Unreported | During breastfeeding | Children with different age group |
|  | | | | | | | | |
| **3. INFANTS** | | | | | | | | |
| **Study, Year** | **n** | **Population who received vitamin D** | **Type of vitamin D administered** | **Route of administration and forms** | | **Doses of vitamin D administered** | **Time of administration of vitamin D** | **Time of follow-up for outcome(s)** |
| ***Randomized control trials*** | | | | | | | | |
| Alonso, 2011 | 102* | Full-term 1-month-old healthy **infants** | Vitamin D (cholecalciferol) | Oral (drops) | | 402 IU/day | 12 months | At 1 year of age |
| Chan, 1982 | 51* | Breastfeeding healthy term **infants** and **lactating mother** | Vitamin D | Unreported | | 400 IU/day | 4 months | At 1 year of age |
| Greer, 1982 | 18* | Breastfeeding healthy term **infants** | Vitamin D | Unreported | | 400 IU/day | Until infants were weaned from breast-feeding | At 6 months of age |
| Madar, 2009 | 66*** | 6 week-old **infants** | Vitamin D2 | Oral (drops) | | 400 IU/day | 6 weeks | At 3 months of age |
| Ponnapakkam, 2010 | 80* | Breast-fed **term infants**† | Vitamin D3 | Oral (drops) | | 200 IU/day | Between 4 to 6 months | At 6 months of age |
| ***Non-randomized studies*** | | | | | | | | |
| Allen, 2013 | 2252 | **Infants** | Vitamin D supplement | Unreported) | | Unreported | During infancy | Infants with aged between 11 and 15 months |
| Anderson 2015 | 2478 | **Infants** | Vitamin D supplement | Unreported | | 400 IU/day | During childhood | Infants with aged between 0 to 5 years |
| Hypponen, 2004 | 7648 | **Infants** | Vitamin D supplement | Unreported | | <2000 IU to >2000 IU‡ | During the first year of life | At age 31 years |
| * Patients randomized. ** Results presented in metanalysis correspond to pooled data. Single oral dose was administered as cholecalciferol (oral bolus). *** Randomization was carried out at the health clinic level. † Two group of infants received vitamin D: one group started the vitamin D supplementation at birth and another group started at 2 months of age. ‡The daily dose of vitamin D was calculated on the basis of concentration of vitamin D in the product used and the reported dosage (categorized as <50 μg, 50 μg, or >50 μg). | | | | | | | | |
