# Supplementary material for: World Allergy Organization-McMaster University Guidelines for Allergic Disease Prevention (GLAD-P): Vitamin D
Source: World Allergy Organ J. 2016 May 17;9:17. doi: 10.1186/s40413-016-0108-1 (PMC4869275; doi:10.1186/s40413-016-0108-1)
Supplement: Additional file 4: — Evidence profiles. (DOCX 79 kb) [file 40413_2016_108_MOESM4_ESM.docx]

**Online supplement 4. Evidence profiles**

**EVIDENCE PROFILE: *PREGNANCY WOMEN***

**Authors**: Juan José Yepes-Nuñez, Jan Brozek, Carlos Cuello-García, Yuan Zhang, Gian Paolo Morgano, Holger Schünemann
**Date**: January 19, 2015; updated August 2015

**Question**: Vitamin D compared to no vitamin D in **pregnant women** for the prevention of allergies in their children
**Setting**: General population
**Bibliography**: Allen 2013 ([1](#_ENREF_1)), Anderson 2015 ([1](#_ENREF_1)), Brooke 1980 ([2](#_ENREF_2)), Goldring 2013 ([3](#_ENREF_3)) , Hossain 2014 ([4](#_ENREF_4)), Mallet 1986 ([5](#_ENREF_5)), Roth 2013 ([6](#_ENREF_6), [7](#_ENREF_7)), Sablok ([8](#_ENREF_8)), Yu 2009 ([9](#_ENREF_9))

| **Quality assessment** | | | | | | | **№ of patients** | | **Effect** | | **Quality** | **Importance** |
| --- | --- | --- | --- | --- | --- | --- | --- | --- | --- | --- | --- | --- |
| **№ of studies** | **Study design** | **Risk of bias** | **Inconsistency** | **Indirectness** | **Imprecision** | **Other considerations** | **Vitamin D** | **no intervention/placebo** | **Relative (95% CI)** | **Absolute (95% CI)** |  |  |
| **Eczema (follow up: 3 years)** | | | | | | | | | | | | |
| 1 | randomised trials | serious ^1^ | not serious | not serious ^2^ | very serious ^3^ | none | 30/120 (25.0%) | 15/49 (30.6%) | **RR 0.96** (0.57 to 1.61) | 12 fewer per 1000 (from 132 more to 187 fewer) | ⨁◯◯◯ VERY LOW | CRITICAL |
|  |  |  |  |  |  |  |  | 1.0% ^4^ |  | 0 fewer per 1000 (from 4 more to 6 fewer) |  |  |
| **Allergic rhinitis (follow up: 3 years)** | | | | | | | | | | | | |
| 1 | randomised trials | serious ^1^ | not serious | not serious ^2^ | very serious ^5^ | none | 11/101 (10.9%) | 7/49 (14.3%) | **RR 0.76** (0.31 to 1.85) | 34 fewer per 1000 (from 99 fewer to 121 more) | ⨁◯◯◯ VERY LOW | CRITICAL |
|  |  |  |  |  |  |  |  | 14.6% ^6^ |  | 35 fewer per 1000 (from 101 fewer to 124 more) |  |  |
| **Asthma/wheezing (follow up: 3 years)** | | | | | | | | | | | | |
| 1 | randomised trials | serious ^1^ | not serious | not serious ^2^ | very serious ^7^ | none | 17/108 (15.7%) | 7/50 (14.0%) | **RR 1.12** (0.50 to 2.54) ^8^ | 17 more per 1000 (from 70 fewer to 216 more) | ⨁◯◯◯ VERY LOW | CRITICAL |
|  |  |  |  |  |  |  |  | 9.4% ^9^ |  | 11 more per 1000 (from 47 fewer to 145 more) |  |  |
| **Food allergy (follow up: 3 years)** | | | | | | | | | | | | |
| 1 | randomised trials | serious ^1^ | not serious | not serious ^2^ | very serious ^10^ | none | 12/102 (11.8%) | 3/49 (6.1%) | **RR 1.92** (0.57 to 6.50) ^12^ | 56 more per 1000 (from 26 fewer to 337 more) | ⨁◯◯◯ VERY LOW | CRITICAL |
|  |  |  |  |  |  |  |  | 3.3% ^11^ |  | 30 more per 1000 (from 14 fewer to 182 more) |  |  |
| **Rickets** | | | | | | | | | | | | |
| 1 | randomised trials | not serious | not serious | not serious | very serious ^13^ | none | 0/59 (0.0%) | 0/67 (0.0%) | not estimable | not estimable | ⨁⨁◯◯ LOW | CRITICAL |
| **Nutritional status: birth weight (gr)** | | | | | | | | | | | | |
| 6 | randomised trials | not serious | not serious | not serious | serious ^16^ | none | 455 | 366 | - | MD **52.78 more** (64.34 fewer to 169.9 more) | ⨁⨁⨁◯ MODERATE | CRITICAL |
| **Nutrition status at 1 year (follow up: mean 1 years; assessed with: weight at 1 year [gr])** | | | | | | | | | | | | |
| 1 | randomised trials | not serious | not serious | serious ^14^ | serious ^15^ | none | 66 | 69 | - | MD **100 more** (273.48 fewer to 473.48 more) | ⨁⨁◯◯ LOW | CRITICAL |
| **Adverse effects: gestational age (weeks)** | | | | | | | | | | | | |
| ­­­4 | randomised trials | not serious | not serious | not serious | serious ^16^ | none | 326 | 280 | - | MD **0.43 weeks fewer** (0.88 fewer to 0.02 more) | ⨁⨁⨁◯ MODERATE | CRITICAL |
| **Adverse effects: small for gestational age (assessed with: weight lower than 10th centile or less than 2.5 kg)** | | | | | | | | | | | | |
| 3 | randomised trials | serious ^17^ | not serious | not serious | very serious ^18^ | none | 37/253 (14.6%) | 48/213 (22.5%) | **RR 0.67** (0.37 to 1.19) | 74 fewer per 1000 (from 142 fewer to 43 more) | ⨁◯◯◯ VERY LOW | CRITICAL |
| **Any adverse events (children)** | | | | | | | | | | | | |
| 2 | randomised trials | not serious | not serious | not serious | very serious ^13^ | none | 0/117 (0.0%) | 0/117 (0.0%) | not estimable | 0 fewer per 1000 (from 20 more to 20 fewer) | ⨁⨁◯◯ LOW | CRITICAL |
| **Serious adverse events (children)** | | | | | | | | | | | | |
| 3 | randomised trials | not serious | not serious | not serious | very serious ^5^ | none | 8/213 (3.8%) | 10/213 (4.7%) | **RR 0.79**  (0.33 to 1.90) | 10 fewer per 1000 (from 31 fewer to 42 more) | ⨁⨁◯◯ LOW | CRITICAL |
| **Symptomatic Hypocalcaemia** | | | | | | | | | | | | |
| 1 | randomised trials | serious ^1^ | not serious | not serious | very serious ^19^ | none | 0/59 (0.0%) | 5/67% (7.5%) | **RR 0.10** (0.01 to 1.82**)** | 67 fewer per 1000 (from 61 more to 74 fewer) | ⨁◯◯◯ VERY LOW | CRITICAL |
| **Any adverse events (mother) (assessed with: Preeclampsia)** | | | | | | | | | | | | |
| 3 | randomised trials | serious ^20^ | not serious | not serious | very serious ^21^ | none | 36/254 (14.2%) | 31/266 (11.7%) | **RR 1.46** (0.31 to 6.78) | 54 more per 1000 (from 80 fewer to 674 more) | ⨁◯◯◯ VERY LOW | CRITICAL |
| **Serious adverse events (mother) ^22^** | | | | | | | | | | | | |
| 2 | randomised trials | not serious | not serious | not serious | serious ^23^ | none | 12/8158 (7.6%) | 8/163 (4.9%) | **RR 1.55** (0.65 to 3.69) | 27 more per 1000 (from 17 fewer to 132 more) | ⨁⨁◯◯  LOW | CRITICAL |

MD – mean difference, RR – relative risk

1. Probably no concealment of allocation and no blinding of participants and caregivers.
2. Only 3 years of observation.
3. Only 45 events; confidence interval does not exclude an appreciable benefit or an appreciable harm.
4. Range of prevalence of eczema in ISAAC Phase Three ([10](#_ENREF_10))
5. Only 18 events; confidence interval does not exclude an appreciable benefit or an appreciable harm.
6. Global total prevalence of rhinoconjunctivitis symptoms for the 13- to 14-year old children according with Phase Three ISAAC (14.6%) ([11](#_ENREF_11))
7. Only 24 events; confidence interval does not exclude an appreciable benefit or an appreciable harm.
8. In the cohort study ([1](#_ENREF_1)) vitamin D was associated with low risk of developing wheezing (OR: 0.65, 95% CI: 0.46 to 0.93)
9. Global total prevalence of asthma symptoms in the 6-7 year age according with Phase Three ISAAC in 9.4%([12](#_ENREF_12))
10. Only 15 events; confidence interval does not exclude an appreciable benefit or an appreciable harm.
11. Prevalence in the population based on food challenge tests to any food according with meta-analysis of Rona et al. 2007 ([13](#_ENREF_13))
12. This result is consistent with the result of the only observational study ([14](#_ENREF_14)) that reported this outcome that also found potentially increased risk (OR: 1.50, 95% CI: 0.78 to 2.88)
13. No events in a small study.
14. There is uncertainty to what extent weight at 1 year of age represents a nutritional status.
15. Only 135 children.
16. Confidence interval does not exclude an appreciable benefit or an appreciable harm.
17. Neither method of randomization nor allocation concealment were described in both studies.
18. Only 65 events. confidence interval does not exclude an appreciable benefit or an appreciable harm
19. Only 5 events; confidence interval does not exclude an appreciable benefit or an appreciable harm
20. Only 2 of 6 studies reported this outcome.
21. Only 20 events; confidence interval does not exclude an appreciable benefit or an appreciable harm
22. Only 2 of 3 studies ([4](#_ENREF_4), [6](#_ENREF_6)) reported serious adverse events
23. Only 20 events; confidence interval does not exclude an appreciable benefit or an appreciable harm

**EVIDENCE PROFILE: *BREASTFEEDING WOMEN***

**Authors**: Juan José Yepes-Nuñez, Jan Brozek, Carlos Cuello-García, Yuan Zhang, Gian Paolo Morgano, Holger Schünemann
**Date**: January 19, 2015; updated August 2015
**Question**: Vitamin D compared to no vitamin D in **breastfeeding women** for the prevention of allergies in their children
**Setting**: General population
**Bibliography**:, Bener 2012 ([15](#_ENREF_15)), Rothberg 1982 ([16](#_ENREF_16))

| **Quality assessment** | | | | | | | **№ of patients** | | **Effect** | | **Quality** | **Importance** |
| --- | --- | --- | --- | --- | --- | --- | --- | --- | --- | --- | --- | --- |
| **№ of studies** | **Study design** | **Risk of bias** | **Inconsistency** | **Indirectness** | **Imprecision** | **Other considerations** | **Vitamin D** | **no intervention/placebo** | **Relative (95% CI)** | **Absolute (95% CI)** |  |  |
| **Asthma/wheezing (follow-up: up to 16 years)** | | | | | | | | | | | | |
| 1 | observational studies | serious ^1^ | not serious | not serious ^2^ | serious ^3^ | none | 483 cases 483 controls | | **OR 1.09** (0.84 to 1.40) | - | ⨁◯◯◯ VERY LOW | CRITICAL |
|  |  |  |  |  |  |  | - | 9.4% ^4^ |  | 8 more per 1000 (from 14 fewer to 33 more) |  |  |
| **Allergic rhinitis – not measured** | | | | | | | | | | | | |
| - | - | - | - | - | - | - | - | - | – | - | - | CRITICAL |
| **Eczema – not measured** | | | | | | | | | | | | |
| - | - | - | - | - | - | - | - | - | – | - | - | CRITICAL |
| **Food allergy – not measured** | | | | | | | | | | | | |
| - | - | - | - | - | - | - | - | - | – | - | - | CRITICAL |
| **Any allergy** | | | | | | | | | | | | |
| - | - | - | - | - | - | - | - | - | – | - | - | CRITICAL |
| **Rickets (follow-up: up to 6 weeks)** | | | | | | | | | | | | |
| 1 | randomised trials | serious ^5^ | not serious | not serious | very serious ^6^ | none | 0/18 (0.0%) | 0/10 (0.0%) | not estimable | 0 fewer per 1000 (from 140 more to 140 fewer) | ⨁◯◯◯ VERY LOW | CRITICAL |
| **Adverse effects** | | | | | | | | | | | | |
| - | - | - | - | - | - | - | - | - | – | - | - | CRITICAL |

MD – mean difference, RR – relative risk

1. Controls were sampled from a population that is unlikely to be representative of the population from which cases were selected; analysis was not adjusted for confounding factors.
2. Study was conducted among children in Qatar of whom over 80% had vitamin D deficiency.
3. 95% confidence interval does not exclude an appreciable benefit or appreciable harm.
4. Global total prevalence of asthma symptoms in the 6-7 year age according with Phase Three ISAAC ([12](#_ENREF_12))
5. Probably no concealment of allocation and no blinding of participants and caregivers.
6. No events and 95% confidence interval does not exclude an appreciable benefit or appreciable harm.

**EVIDENCE PROFILE: *INFANTS***

**Authors**: Juan José Yepes-Nuñez, Jan Brozek, Carlos Cuello-García, Yuan Zhang, Gian Paolo Morgano, Holger Schünemann
**Date**: January 19, 2015; updated August 2015
**Question**: Vitamin D compared to no vitamin D in **infants** for the prevention of allergies
**Setting**: General population
**Bibliography**: Allen 2013 ([14](#_ENREF_14)), Alonso 2011 ([17](#_ENREF_17)), Anderson 2015 ([1](#_ENREF_1)), Chan 1982 ([18](#_ENREF_18)), Greer 1982 ([19](#_ENREF_19)), Hypponen 2004 ([20](#_ENREF_20)), Madar ([21](#_ENREF_21)), Ponnapakkam ([22](#_ENREF_22))

| **Quality assessment** | | | | | | | **№ of patients** | | | **Effect** | | **Quality** | **Importance** |
| --- | --- | --- | --- | --- | --- | --- | --- | --- | --- | --- | --- | --- | --- |
| **№ of studies** | **Study design** | **Risk of bias** | **Inconsistency** | **Indirectness** | **Imprecision** | **Other considerations** | **Vitamin D** | | **No intervention/placebo** | **Relative (95% CI)** | **Absolute (95% CI)** |  |  |
| **Allergic rhinitis (follow up: 31 years)** | | | | | | | | | | | | | |
| 1 | observational studies | serious ^1^ | not serious | not serious | serious ^2^ | none | 1974/6748 (29.3%) | | 3/20 (15.0%) | **RR 1.95** (0.69 to 5.54) | 143 more per 1000 (from 47 fewer to 681 more) | ⨁◯◯◯ VERY LOW | CRITICAL |
|  |  |  |  |  |  |  |  |  | 14.6% ^16^ |  | 139 more per 1000 (from 45 fewer to 663 more) |  |  |
| **Asthma/wheezing (follow up: 31 years)** | | | | | | | | | | | | | |
| 1 | observational studies | serious ^1^ | not serious | not serious | serious ^3^ | none | 470/6748 (7.0%) | | 0/20 (0.0%) | **RR 3.07** (0.19 to 50.88) | 0 fewer per 1000 (from 0 fewer to 0 fewer) | ⨁◯◯◯ VERY LOW | CRITICAL |
|  |  |  |  |  |  |  |  |  | 9.4% ^17^ |  | 195 more per 1000 (from 76 fewer to 1000 more) |  |  |
| **Asthma/wheezing (follow up: up to 72 months)** | | | | | | | | | | | | | |
| 1 | observational studies | not serious | not serious | not serious | serious ^15^ | none | | 493/2478 (19.9%) | 9.4% ^17^ | **RR 1.00** (0.081 to 1.23) | 0 fewer per 1000 (from 16 fewer to 19 more) | ⨁◯◯◯ VERY LOW | CRITICAL |
| **Food allergy (follow up: 1 years)** | | | | | | | | | | | | | |
| 1 | observational studies | serious ^4^ | not serious | serious ^5^ | serious ^6^ | none | 21/274 (7.7%) | | 30/207 (14.5%) | **OR 0.49** (0.27 to 0.88) | 68 fewer per 1000 (from 15 fewer to 101 fewer) | ⨁◯◯◯ VERY LOW | CRITICAL |
|  |  |  |  |  |  |  |  |  | 3.3% ^18^ |  | 17 fewer per 1000 (from 4 fewer to 24 fewer) |  |  |
| **Eczema – not measured** | | | | | | | | | | | | | |
| - | - | - | - | - | - | - | - | | - | – | - | - | CRITICAL |
| **Rickets (follow up: range 2 months to 12 months)** | | | | | | | | | | | | | |
| 4 | randomised trials | serious ^7^ | not serious | serious ^8^ | serious ^9^ | none | 0/86 (0.0%) | | 0/91 (0.0%) | not estimable | 0 fewer per 1000 (from 40 more to 40 fewer) | ⨁◯◯◯ VERY LOW | CRITICAL |
| **Nutritional status (body weight in grams) (follow up: range 6 months to 12 months)** ^19^ | | | | | | | | | | | | | |
| 1 | randomised trials | serious ^10^ | not serious | not serious | serious ^11^ | none | 36 | | 44 | There were no significant differences in weight between arms ^19^ | | ⨁⨁◯◯ LOW | CRITICAL |
| **Any adverse events** ^20^ | | | | | | | | | | | | | |
| 3 | randomised trials | serious ^12^ | not serious | not serious | very serious ^13^ | none | 4/58 (6.9%) | | 4/51 (7.8%) | **RR 0.85** (0.23 to 3.14) | 12 fewer per 1000 (from 60 fewer to 168 more) | ⨁◯◯◯ VERY LOW | CRITICAL |
| **Serious adverse events** | | | | | | | | | | | | | |
| 1 | randomised trials | serious ^14^ | not serious | not serious | very serious ^15^ | none | 0/17 (0.0%) | | 1/8 (12.5%) | **RR 0.17** (0.01 to 3.70) | 104 fewer per 1000 (from 124 fewer to 338 more) | ⨁◯◯◯ VERY LOW | CRITICAL |

MD – mean difference, RR – relative risk

1. Criteria for diagnosis of asthma and allergic rhinitis were not provided; results are not adjusted for confounding factors despite “cohort members with family history of asthma were less likely to receive supplementation according to recommendations (...) and many of the same characteristics that were predictive of worse compliance were associated with reduced risk of allergies”.
2. Only 3 events in a very small control group make the resulrs very fragile; confidence interval does not exclude an appreciable benefit or an appreciable harm. Study authors combined the group that never received vitamin D with a group that used it irregularly to reduce fragility of the results; if those using vitamin D regularly were compared with those who either did not use it or used it irregularly the RR would be 1.31 (95% CI: 1.15 to 1.49).
3. There were no events in a very small control group; confidence interval does not exclude an appreciable benefit or an appreciable harm. Study authors combined the group that never received vitamin D with a group that used it irregularly to reduce fragility of the results; if those using vitamin D regularly were compared with those who either did not use it or used it irregularly the RR would be 1.33 (95% CI: 1.00 to 1.79).
4. Results were not adjusted for confounding factors.
5. Outcome was measured after 1 year of life, which is likely too short to reliably measure development of food allergy.
6. Only 51 events.
7. Probably no blinding of participants and caregivers in three of four studies.
8. Follow-up was 2 to 12 months which is not long enough for assessing development of rickets.
9. No events among 177 children; 95% confidence interval does not exclude an appreciable clinical benefit or no difference.
10. Random sequence generation was not described – likely quasi-random method of allocation was used.
11. 95% confidence interval does not exclude an appreciable clinical benefit or no difference.
12. Unclear random sequence generation and blinding in one study and unclear allocation concealment in two studies.
13. Only 8 events; confidence interval does not exclude an appreciable harm.
14. Unclear random sequence generation and blinding.
15. Confidence interval does not exclude an appreciable harm.
16. Global total prevalence of rhinoconjunctivitis symptoms for the 13- to 14-year old children according with Phase Three ISAAC (14.6%) ([11](#_ENREF_11)).
17. Global total prevalence of asthma symptoms in the 6-7 year age according with Phase Three ISAAC ([12](#_ENREF_12)).
18. Prevalence in the population based on food challenge tests to any food according with meta-analysis of Rona et al. 2007 ([13](#_ENREF_13)).
19. One study ([18](#_ENREF_18)) with large loss to follow-up reported nutritional status (weight) but vitamin D supplementation was provided to infants and their breastfeeding mothers; data available only from graph with no variability; point estimates suggest no difference between the groups.
20. Two additional studies reported adverse events in population aged 1 to 5 years old [Marchisio 2013 ([23](#_ENREF_23))] and 2 to 6 years old [Hower 2013 ([24](#_ENREF_24))]. Marchisio 2013 described that treatment was well tolerated, and the parents of the children receiving vitamin D reported no significant adverse event. Hower 2013 reported 85 AEs (e.g., conjunctivitis, viral infection, abdominal pain, constipation, viral rash, dermatitis, cuts and bruises, etc.). There were two SAEs, one in each group, with complete recovery (cyanosis due to croup, Rota-positive gastroenteritis). None of the AEs was considered to have been related to the study product, and there were no differences between study groups.

**REFERENCES**

1. Anderson LN, Chen Y, Omand JA, Birken CS, Parkin PC, To T, et al. Vitamin D exposure during pregnancy, but not early childhood, is associated with risk of childhood wheezing. Journal of developmental origins of health and disease. 2015;6(4):308-16.

2. Brooke OG, Brown IR, Bone CD, Carter ND, Cleeve HJ, Maxwell JD, et al. Vitamin D supplements in pregnant Asian women: effects on calcium status and fetal growth. British medical journal. 1980;280(6216):751-4.

3. Goldring ST, Griffiths CJ, Martineau AR, Robinson S, Yu C, Poulton S, et al. Prenatal vitamin d supplementation and child respiratory health: a randomised controlled trial. PloS one. 2013;8(6):e66627.

4. Hossain N, Kanani FH, Ramzan S, Kausar R, Ayaz S, Khanani R, et al. Obstetric and neonatal outcomes of maternal vitamin D supplementation: results of an open-label, randomized controlled trial of antenatal vitamin D supplementation in Pakistani women. The Journal of clinical endocrinology and metabolism. 2014;99(7):2448-55.

5. Mallet E, Gugi B, Brunelle P, Henocq A, Basuyau JP, Lemeur H. Vitamin D supplementation in pregnancy: a controlled trial of two methods. Obstetrics and gynecology. 1986;68(3):300-4.

6. Roth DE, Al Mahmud A, Raqib R, Akhtar E, Perumal N, Pezzack B, et al. Randomized placebo-controlled trial of high-dose prenatal third-trimester vitamin D3 supplementation in Bangladesh: the AViDD trial. Nutrition journal. 2013;12:47.

7. Roth DE, Perumal N, Al Mahmud A, Baqui AH. Maternal vitamin D3 supplementation during the third trimester of pregnancy: effects on infant growth in a longitudinal follow-up study in Bangladesh. The Journal of pediatrics. 2013;163(6):1605-11 e3.

8. Sablok A, Thariani K, Batra A, Bharti R, Aggarwal AR, Kabi BC, et al. Supplementation of Vitamin D in pregnancy and its correlation with feto-maternal outcome. Clinical endocrinology. 2015; 83(4):536-41.

9. Yu CK, Sykes L, Sethi M, Teoh TG, Robinson S. Vitamin D deficiency and supplementation during pregnancy. Clin Endocrinol (Oxf). 2009;70(5):685-90.

10. Odhiambo JA, Williams HC, Clayton TO, Robertson CF, Asher MI, Group IPTS. Global variations in prevalence of eczema symptoms in children from ISAAC Phase Three. The Journal of allergy and clinical immunology. 2009;124(6):1251-8 e23.

11. Ait-Khaled N, Pearce N, Anderson HR, Ellwood P, Montefort S, Shah J, et al. Global map of the prevalence of symptoms of rhinoconjunctivitis in children: The International Study of Asthma and Allergies in Childhood (ISAAC) Phase Three. Allergy. 2009;64(1):123-48.

12. Lai CK, Beasley R, Crane J, Foliaki S, Shah J, Weiland S, et al. Global variation in the prevalence and severity of asthma symptoms: phase three of the International Study of Asthma and Allergies in Childhood (ISAAC). Thorax. 2009;64(6):476-83.

13. Rona RJ, Keil T, Summers C, Gislason D, Zuidmeer L, Sodergren E, et al. The prevalence of food allergy: a meta-analysis. The Journal of allergy and clinical immunology. 2007;120(3):638-46.

14. Allen KJ, Koplin JJ, Ponsonby AL, Gurrin LC, Wake M, Vuillermin P, et al. Vitamin D insufficiency is associated with challenge-proven food allergy in infants. The Journal of allergy and clinical immunology. 2013;131(4):1109-16, 16 e1-6.

15. Bener A, Ehlayel MS, Tulic MK, Hamid Q. Vitamin D deficiency as a strong predictor of asthma in children. International archives of allergy and immunology. 2012;157(2):168-75.

16. Rothberg AD, Pettifor JM, Cohen DF, Sonnendecker EW, Ross FP. Maternal-infant vitamin D relationships during breast-feeding. The Journal of pediatrics. 1982;101(4):500-3.

17. Alonso A, Rodriguez J, Carvajal I, Prieto MA, Rodriguez RM, Perez AM, et al. Prophylactic vitamin D in healthy infants: assessing the need. Metabolism: clinical and experimental. 2011;60(12):1719-25.

18. Chan GM, Roberts CC, Folland D, Jackson R. Growth and bone mineralization of normal breast-fed infants and the effects of lactation on maternal bone mineral status. The American journal of clinical nutrition. 1982;36(3):438-43.

19. Greer FR, Searcy JE, Levin RS, Steichen JJ, Steichen-Asche PS, Tsang RC. Bone mineral content and serum 25-hydroxyvitamin D concentrations in breast-fed infants with and without supplemental vitamin D: one-year follow-up. The Journal of pediatrics. 1982;100(6):919-22.

20. Hypponen E, Sovio U, Wjst M, Patel S, Pekkanen J, Hartikainen AL, et al. Infant vitamin d supplementation and allergic conditions in adulthood: northern Finland birth cohort 1966. Annals of the New York Academy of Sciences. 2004;1037:84-95.

21. Madar AA, Klepp KI, Meyer HE. Effect of free vitamin D(2) drops on serum 25-hydroxyvitamin D in infants with immigrant origin: a cluster randomized controlled trial. European journal of clinical nutrition. 2009;63(4):478-84.

22. Ponnapakkam T, Bradford E, Gensure R. A treatment trial of vitamin D supplementation in breast-fed infants: universal supplementation is not necessary for rickets prevention in Southern Louisiana. Clinical pediatrics. 2010;49(11):1053-60.

23. Marchisio P, Consonni D, Baggi E, Zampiero A, Bianchini S, Terranova L, et al. Vitamin D supplementation reduces the risk of acute otitis media in otitis-prone children. The Pediatric infectious disease journal. 2013;32(10):1055-60.

24. Hower J, Knoll A, Ritzenthaler KL, Steiner C, Berwind R. Vitamin D fortification of growing up milk prevents decrease of serum 25-hydroxyvitamin D concentrations during winter: a clinical intervention study in Germany. European journal of pediatrics. 2013;172(12):1597-605.
