# Supplementary material for: World Allergy Organization-McMaster University Guidelines for Allergic Disease Prevention (GLAD-P): Vitamin D
Source: World Allergy Organ J. 2016 May 17;9:17. doi: 10.1186/s40413-016-0108-1 (PMC4869275; doi:10.1186/s40413-016-0108-1)
Supplement: Additional file 5: — Evidence to Decision table. (DOC 196 kb) [file 40413_2016_108_MOESM5_ESM.doc]

**Additional file 5. Evidence to Decision table**

**Evidence to Decision table: *Pregnant women***

| **Should vitamin D be used in pregnant women for the prevention of allergy in their children?** | |
| --- | --- |
| ***Population:*** pregnant women and their children  ***Intervention:*** vitamin D  ***Comparison:*** no vitamin D  ***Main outcomes:*** development of allergy, nutritional status, adverse effects  ***Setting:*** outpatient  ***Perspective:*** individual patient | ***Background:*** Allergic diseases represent a spectrum of health conditions and a worldwide burden in different populations. In infants, its prevalence depends highly on the allergic status of their parents, being approximately of 10% in those without an allergic parent or sibling, versus 20% to 30% in those with an atopic background in their first-degree relatives. Allergic conditions (asthma, rhinitis, eczema, food allergy) are considered an economic burden that can also affects the quality of life of affected populations.  The associations between vitamin D concentrations and various conditions and diseases have been assessed in a large and rapidly expanding literature. In addition to observational studies, numerous randomised trials have examined the effect of vitamin D supplementation on a range of outcomes. There is emerging evidence that vitamin D plays a role in the immune system that has not yet been clearly described. Vitamin D has been reported to modulate immune functioning in cell culture and animal models. Vitamin D, specifically its active form, calcitriol is a regulator of both adaptive and innate immune responses. However, its role is complex and not fully understood. |

|  | **Criteria** | **Judgements** | **Research evidence** | **Additional considerations** |
| --- | --- | --- | --- | --- |
| Problem | **Is there a problem priority?** | ○ No  ○ Probably no  ○ Uncertain  ○ Probably yes  **X Yes**  ○ Varies | Baseline risks for development of allergy in children are high. In the included studies it was 12% to 17% over 3 to 5 years. |  |
| Benefits & harms of the options | **What is the overall certainty of this evidence?** | ○ No included studies  **X Very low**  ○ Low  ○ Moderate  ○ High | **Values and preferences**  **Outcome**  **Value placed on an outcome (utility) ^1^**  **Variability**  **Certainty of the evidence (GRADE)**  **Atopic Dermatitis / Eczema ^2^**  **-** Non-recurrence of eczema  - Disease controlled state  - Mild atopic eczema  - Moderate atopic eczema  1  0.98  0.86  0.69  VERY LOW  **Allergic Rhinitis**  - No symptoms  - Mild  - Moderate  - Severe allergic rhinitis & mild allergic asthma  - Severe allergic rhinitis and moderate to  - Severe allergic asthma  0.78  0.76  0.74  0.73  0.70  VERY LOW  **Asthma/ Wheezing ^3,4^**  - Mild symptoms  - Moderate symptoms  - Severe symptoms  0.55; 0.79  0.49; 0.7  0.16; 0.28  VERY LOW  **Food Allergy ^2^**  0.9  VERY LOW  **Adverse Events**  -  **Nutrition Status**  -  **Rickets**  -  1 Utility is expressed on a scale from 0 (death) to 1 (perfect health)  2 Measured with HUI  3 Utilities derived from days with symptoms, for both adults and children  4 Measured with VAS and SG | The concomitant presence of AR and asthma disrupted the ability to get a good night's sleep (79%), to participate in leisure and sports activities (75%), to concentrate at work or school (69% of adults, 73% of children), and to enjoy social activities (57% of adults, 51% of children). Due to these diseases, many (56%) patients avoided the outdoors during the allergy season because of worsening asthma symptoms.  Qualitative studies revealed the parental experience and patient experience related to children/teenagers with food allergy. The experiences related to food allergy include: experience of isolation or exclusion or feeling of being constrained, living with risks, adjustment due to disease for both parents and children; emotional tension between family members, frustration and difficulties due to untimely diagnosis and recommendations for food allergy for parents; as well as difficulties to try new food, perceiving parental control as protective for children/teenagers.  Acronym:  HUI: Health Utility Index; SG: Standard Gamble; TTO: Time Trade Off; VAS: Visual Analogue Scale.  Due to limited and inconsistent reports, the panel members considered there are possible important uncertainty and probable variability around the estimates on values and preferences.  The definition of “Vitamin D sufficiency/insufficiency”, time of supplementation during pregnancy, setting, food fortification, as well as natural environments, all increase the complexity of addressing this research question. |
|  | **Is there important uncertainty about how much people value the main outcomes?** | ○ Important uncertainty or variability  ○ Possibly important uncertainty or variability  ○ Probably no important uncertainty of variability  ○ No important uncertainty of variability  ○ No known undesirable  **X Possibly important uncertainty and probably variability** |  |  |
|  | **Are the desirable anticipated effects large?** | ○ No  **X Probably no**  ○ Uncertain  ○ Probably yes  ○ Yes  ○ Varies |  |  |
|  | **Are the undesirable anticipated effects small?** | ○ No  ○ Probably no  ○ Uncertain  **X Probably yes**  ○ Yes  ○ Varies |  |  |
|  | **Are the desirable effects large relative to undesirable effects?** | ○ No  **X Probably no**  ○ Uncertain  ○ Probably yes  ○ Yes  ○ Varies |  |  |
| Resource use | **Are the resources required small?** | ○ No  ○ Probably no  ○ Uncertain  **X Probably yes**  ○ Yes  ○ Varies | We searched major medical databases (Medline, EMBASE) and economic databases, as well as the price information available on the Internet, The annual cost for Vitamin D supplementation in the USA ranges from $10.15 to $47.10 for an adult, and from $13.20 to $20.45 for a child. | Price may vary according to the countries, regions, formulations etc. |
|  | **Is the incremental cost small relative to the net benefits?** | ○ No  **X Probably no**  ○ Uncertain  ○ Probably yes  ○ Yes  ○ Varies | No studies including health economic evaluations in relation to specific disease outcomes for vitamin D supplementation during pregnancy. | Although the resources required small, the incremental cost could be large due to “probable no” large desirable effects. |
| Equity | **What would be the impact on health inequities?** | ○ Increased  **X Probably increased**  ○ Uncertain  ○ Probably reduced  ○ Reduced  ○ Varies | No research evidence | In some settings it may be important to consider equity as the access may depend on socioeconomic status of the country or setting where coverage will depend on policymakers.  It may incur equity problems to invest resources on strategies with “probably no” large desirable effects, rather than invest on other effective treatment/prevention options. |
| Acceptability | **Is the option acceptable to key stakeholders?** | ○ No  ○ Probably no  ○ Uncertain  ○ Probably yes  **X Yes**  ○ Varies | No research evidence | Vitamin D supplementation is acceptable. |
| Feasibility | **Is the option feasible to implement?** | ○ No  ○ Probably no  ○ Uncertain  ○ Probably yes  **X Yes**  ○ Varies | No research evidence | Vitamin D supplementation is feasible. |

| **Recommendation**  **Question: Should VITAMIN D vs. no VITAMIN D be used pregnant women for the prevention of allergy in their children?** | | | | | |
| --- | --- | --- | --- | --- | --- |
| **Balance of consequences** | Undesirable consequences *clearly outweigh* desirable consequences in most settings | Undesirable consequences *probably outweigh* desirable consequences in most settings | The balance between desirable and undesirable consequences *is closely balanced or uncertain* | Desirable consequences *probably outweigh* undesirable consequences in most settings | Desirable consequences *clearly outweigh* undesirable consequences in most settings |
|  | ○ | X | ○ | ○ | ○ |

| **Type of recommendation** | We recommend against offering this option | We suggest not offering this option | We suggest offering this option | We recommend offering this option |
| --- | --- | --- | --- | --- |
|  | ○ | X | ○ | ○ |
| **Recommendation** | We suggest that vitamin D supplementation **not be** used in pregnant women (conditional recommendation, very low quality of evidence).  *This recommendation does not apply to pregnant women who have a specific indication for vitamin D supplementation.*  *This will be reviewed and updated as appropriate. Further research will likely have an impact on this recommendation.* | | | |
| **Justification** | Currently there is no evidence suggesting a benefit from supplementation of vitamin D in pregnant women who have a specific indication for vitamin D supplementation. This recommendation is based on very low certainty of evidence, as well as this strategy is not cost-effective and probably increasing health inequities. | | | |
| **Subgroup considerations** | This recommendation for prevention for allergic diseases does not apply to vitamin D deficient women. | | | |
| **Implementation considerations** |  | | | |
| **Monitoring and evaluation** | Incoming studies addressing this research question. | | | |
| **Research possibilities** | Studies are under way that will likely provide more information. | | | |

**Evidence to Decision table: *Breastfeeding women***

| **Should vitamin D be used in breastfeeding women for the prevention of allergy in their children?** | |
| --- | --- |
| ***Population:*** breastfeeding women and their children  ***Intervention:*** vitamin D  ***Comparison:*** no vitamin D  ***Main outcomes:*** development of allergy and rickets  ***Setting:*** outpatient  ***Perspective:*** population | ***Background:*** Allergic diseases represent a spectrum of health conditions and a worldwide burden in different populations. In infants, its prevalence depends highly on the allergic status of their parents, being approximately of 10% in those without an allergic parent or sibling, versus 20% to 30% in those with an atopic background in their first-degree relatives. Allergic conditions (asthma, rhinitis, eczema, food allergy) are considered an economic burden that can also affects the quality of life of affected populations.  The associations between vitamin D concentrations and various conditions and diseases have been assessed in a large and rapidly expanding literature. In addition to observational studies, numerous randomised trials have examined the effect of vitamin D supplementation on a range of outcomes. There is emerging evidence that vitamin D plays a role in the immune system that has not yet been clearly described. Vitamin D has been reported to modulate immune functioning in cell culture and animal models. Vitamin D, specifically its active form, calcitriol is a regulator of both adaptive and innate immune responses. However, its role is complex and not fully understood. |

|  | **Criteria** | **Judgements** | **Research evidence** | **Additional considerations** |
| --- | --- | --- | --- | --- |
| Problem | **Is there a problem priority?** | ○ No ○ Probably no ○ Uncertain ○ Probably yes **X Yes** ○ Varies | Baseline risks for development of allergy in children are high. In the included studies it was 12% to 17% over 3 to 5 years. |  |
| Benefits & harms of the options | **What is the overall certainty of this evidence?** | ○ No included studies **X Very low** ○ Low ○ Moderate ○ High | **Values and preferences**  **Outcome**  **Value placed on an outcome (utility)^1^**  **Variability**  **Certainty of the evidence (GRADE)**  **Atopic Dermatitis / Eczema^2^**  Non-recurrence of eczema  Disease-controlled state  Mild atopic eczema  Moderate atopic eczema  1  0.98  0.86  0.69  VERY LOW  **Allergic Rhinitis**  No symptoms  Mild  Moderate  Severe allergic rhinitis & mild allergic  asthma  Severe allergic rhinitis and moderate to  severe allergic asthma  0.78  0.76  0.74  0.73  0.70  VERY LOW  **Asthma/ Wheezing ^3,4^**  Mild symptoms  Moderate symptoms  Severe symptoms  0.55; 0.79  0.49; 0.7  0.16; 0.28  VERY LOW  **Food Allergy ^2^**  0.9  VERY LOW  **Adverse Events**  -  **Nutrition Status**  -  **Rickets**  -  1 Utility is expressed on a scale from 0 (death) to 1 (perfect health)  2 Measured with HUI  3 Utilities derived from days with symptoms, for both adults and children  4 Measured with VAS and SG  **Summary of findings**  **Outcome**  **Without Vitamin D**  **With Vitamin D**  **Difference (95% CI)**  **Relative effect (RR) (95% CI)**  **Certainty of the evidence**  **(GRADE)**  Asthma/ Wheezing (from observational study)  94 per 1000  **102 per 1000 (80 to 127)**  8 more per 1000 (from 14 fewer to 33 more)  **OR 1.09** (0.84 to 1.4)  ⨁◯◯◯  VERY LOW  Rickets  –  **–**  –  –  –  Atopic Dermatitis / Eczema  –  **–**  –  –  –  Allergic Rhinitis  –  **–**  –  –  –  Food Allergy  –  **–**  –  –  –  Adverse Events  –  **–**  –  –  –  Nutrition Status  –  **–**  –  –  –  Any allergy  –  **–**  –  –  –  Quality of Life  –  **–**  –  –  – | The concomitant presence of AR and asthma disrupted the ability to get a good night's sleep (79%), to participate in leisure and sports activities (75%), to concentrate at work or school (69% of adults, 73% of children), and to enjoy social activities (57% of adults, 51% of children). Due to these diseases, many (56%) patients avoided the outdoors during the allergy season because of worsening asthma symptoms.  Qualitative studies revealed the parental experience and patient experience related to children/teenagers with food allergy. The experiences related to food allergy include: experience of isolation or exclusion or feeling of being constrained, living with risks, adjustment due to disease for both parents and children; emotional tension between family members, frustration and difficulties due to untimely diagnosis and recommendations for food allergy for parents; as well as difficulties to try new food, perceiving parental control as protective for children/teenagers.  Acronym:  HUI: Health Utility Index; SG: Standard Gamble; TTO: Time Trade Off; VAS: Visual Analogue Scale.  Due to limited and inconsistent reports, the panel members considered there are possible important uncertainty and probable variability around the estimates on values and preferences.  The definition of “Vitamin D sufficiency/insufficiency”, time of supplementation, setting, food fortification, as well as natural environments, all increase the complexity of addressing this research question. |
|  | **Is there important uncertainty about how much people value the main outcomes?** | ○ Important uncertainty or variability ○ Possibly important uncertainty or probable variability ○ Probably no important uncertainty of variability ○ No important uncertainty of variability ○ No known undesirable |  |  |
|  | **Are the desirable anticipated effects large?** | ○ No ○ Probably no **X Uncertain** ○ Probably yes ○ Yes ○ Varies |  |  |
|  | **Are the undesirable anticipated effects small?** | ○ No ○ Probably no **X Uncertain** ○ Probably yes ○ Yes ○ Varies |  |  |
|  | **Are the desirable effects large relative to undesirable effects?** | ○ No ○ Probably no **X Uncertain** ○ Probably yes ○ Yes ○ Varies |  |  |
| Resource use | **Are the resources required small?** | ○ No ○ Probably no **X Uncertain** ○ Probably yes ○ Yes ○ Varies | We searched major medical databases (Medline, EMBASE) and economic databases, as well as the price information available on the Internet, The annual cost for Vitamin D supplementation in the USA ranges from $10.15 to $47.10 for an adult, and from $13.20 to $20.45 for a child. | Price may vary according to the countries, regions, formulations etc. |
|  | **Is the incremental cost small relative to the net benefits?** | ○ No ○ Probably no **X Uncertain** ○ Probably yes ○ Yes ○ Varies | No cost-effectiveness analysis related to the preventive use of vitamin D for allergic diseases was identified during the literature review. |  |
| Equity | **What would be the impact on health inequities?** | ○ Increased ○ Probably increased **X Uncertain** ○ Probably reduced ○ Reduced ○ Varies | No research evidence | In some settings it may be important to consider equity as the access may depend on socioeconomic status of the country or setting where coverage will depend on policymakers. |
| Acceptability | **Is the option acceptable to key stakeholders?** | ○ No ○ Probably no ○ Uncertain ○ Probably yes **X Yes** ○ Varies | No research evidence | Vitamin D supplementation is acceptable. |
| Feasibility | **Is the option feasible to implement?** | ○ No ○ Probably no ○ Uncertain ○ Probably yes **X Yes** ○ Varies | No research evidence | Vitamin D supplementation is feasible. |
|  |  |  |  |  |

| **Recommendation**  **Question: Should VITAMIN D vs. no VITAMIN D be used pregnant women for the prevention of allergy in their children?** | | | | | |
| --- | --- | --- | --- | --- | --- |
| **Balance of consequences** | Undesirable consequences *clearly outweigh* desirable consequences in most settings | Undesirable consequences *probably outweigh* desirable consequences in most settings | The balance between desirable and undesirable consequences *is closely balanced or uncertain* | Desirable consequences *probably outweigh* undesirable consequences in most settings | Desirable consequences *clearly outweigh* undesirable consequences in most settings |
|  | ○ | ○ | X | ○ | ○ |

| **Type of recommendation** | We recommend against offering this option | We suggest not offering this option | We suggest offering this option | We recommend offering this option |
| --- | --- | --- | --- | --- |
|  | ○ | X | ○ | ○ |
| **Recommendation** | We suggest that vitamin D supplementation **not be** used in breastfeeding mothers (conditional recommendation, very low quality of evidence).  *This recommendation does not apply to breastfeeding mothers who have a specific indication for vitamin D supplementation.*  *This will be reviewed and updated as appropriate. Further research will likely have an impact on this recommendation.* | | | |
| **Justification** | There is currently no evidence suggesting a benefit from vitamin D supplementation in breastfeeding mothers. However, given the paucity of information any estimate of the effects is very uncertain and does not exclude an appreciable no benefit, no effect or appreciable harm, as well as potential unnecessary cost involved. More studies are necessary for addressing this research question. This recommendation does not include consideration of vitamin D use for secondary prevention for allergic diseases. There may be such studies. | | | |
| **Subgroup considerations** | This recommendation for prevention for allergic diseases does not apply to vitamin D deficient women. | | | |
| **Implementation considerations** | In breastfeeding women already using vitamin D supplementation they should be informed to be able to make an informed choice whether to continue or stop. After reviewing the outcomes, we did not identify the benefits of vitamin D supplementation for breastfeeding women without deficiency. | | | |
| **Monitoring and evaluation** | Incoming studies addressing this research question. | | | |
| **Research possibilities** | RCTs of vitamin D supplementation in breastfeeding women measuring allergy outcomes in children. | | | |

**Evidence to Decision table: *Infants***

| **Should vitamin D be used in infants for the prevention of allergy diseases?** | |
| --- | --- |
| ***Population:*** infants  ***Intervention:*** vitamin D  ***Comparison:*** no vitamin D  ***Main outcomes:*** development of allergy and rickets, nutritional status, adverse events  ***Setting:*** outpatient  ***Perspective:*** individual patient | ***Background:*** Allergic diseases represent a spectrum of health conditions and a worldwide burden in different populations. In infants, its prevalence depends highly on the allergic status of their parents, being approximately of 10% in those without an allergic parent or sibling, versus 20% to 30% in those with an atopic background in their first-degree relatives. Allergic conditions (asthma, rhinitis, eczema, food allergy) are considered an economic burden that can also affects the quality of life of affected populations.  The associations between vitamin D concentrations and various conditions and diseases have been assessed in a large and rapidly expanding literature. In addition to observational studies, numerous randomized trials have examined the effect of vitamin D supplementation on a range of outcomes. There is emerging evidence that vitamin D plays a role in the immune system that has not yet been clearly described. Vitamin D has been reported to modulate immune functioning in cell culture and animal models. Vitamin D, specifically its active form, calcitriol is a regulator of both adaptive and innate immune responses. However, its role is complex and not fully understood. |

|  | **Criteria** | **Judgments** | **Research evidence** | **Additional considerations** |
| --- | --- | --- | --- | --- |
| Problem | **Is there a problem priority?** | ○ No ○ Probably no ○ Uncertain ○ Probably yes **X Yes** ○ Varies | Baseline risks for development of allergy in children are high. In the included studies it was 12% to 17% over 3 to 5 years. |  |
| Benefits & harms of the options | **What is the overall certainty of this evidence?** | ○ No included studies **X Very low** ○ Low ○ Moderate ○ High | **Values and preferences**  **Outcome**  **Value placed on an outcome (utility)^1^**  **Variability**  **Certainty of the evidence (GRADE)**  **Atopic Dermatitis / Eczema ^2^**  Non-recurrence of eczema  Disease-controlled state  Mild atopic eczema  Moderate atopic eczema  1  0.98  0.86  0.69  VERY LOW  **Allergic Rhinitis**  No symptoms  Mild  Moderate  Severe allergic rhinitis & mild allergic  asthma  Severe allergic rhinitis and moderate to  severe allergic asthma  0.78  0.76  0.74  0.73  0.70  VERY LOW  **Asthma/ Wheezing ^3,4^**  Mild symptoms  Moderate symptoms  Severe symptoms  0.55; 0.79  0.49; 0.7  0.16; 0.28  VERY LOW  **Food Allergy ^2^**  0.9  VERY LOW  **Adverse Events**  -  **Nutrition Status**  -  **Rickets**  -  1 Utility is expressed on a scale from 0 (death) to 1 (perfect health)  **2** Measured with HUI  **3** Utilities derived from days with symptoms, for both adults and children  4 Measured with VAS and SG  **Summary of findings**  (See evidence profile below) | The concomitant presence of AR and asthma disrupted the ability to get a good night's sleep (79%), to participate in leisure and sports activities (75%), to concentrate at work or school (69% of adults, 73% of children), and to enjoy social activities (57% of adults, 51% of children). Due to these diseases, many (56%) patients avoided the outdoors during the allergy season because of worsening asthma symptoms.  Qualitative studies revealed the parental experience and patient experience related to children/teenagers with food allergy. The experiences related to food allergy include: experience of isolation or exclusion or feeling of being constrained, living with risks, adjustment due to disease for both parents and children; emotional tension between family members, frustration and difficulties due to untimely diagnosis and recommendations for food allergy for parents; as well as difficulties to try new food, perceiving parental control as protective for children/teenagers.  Due to limited and inconsistent reports, the panel members considered there are possible important uncertainty and probable variability around the estimates on values and preferences.  The definition of “Vitamin D sufficiency/insufficiency”, time of supplementation during pregnancy, setting, food fortification, as well as natural environments, all increase the complexity of addressing this research question. |
|  | **Is there important uncertainty about how much people value the main outcomes?** | ○ Important uncertainty or variability **X Possibly important uncertainty or probable variability** ○ Probably no important uncertainty of variability ○ No important uncertainty of variability ○ No known undesirable |  |  |
|  | **Are the desirable anticipated effects large?** | ○ No ○ Probably no **X Uncertain** ○ Probably yes ○ Yes ○ Varies |  |  |
|  | **Are the undesirable anticipated effects small?** | ○ No ○ Probably no **X Uncertain** ○ Probably yes ○ Yes ○ Varies |  |  |
|  | **Are the desirable effects large relative to undesirable effects?** | ○ No ○ Probably no **X Uncertain** ○ Probably yes ○ Yes ○ Varies |  |  |
| Resource use | **Are the resources required small?** | ○ No ○ Probably no ○ Uncertain ○ Probably yes **X Yes** ○ Varies | We searched major medical databases (Medline, EMBASE) and economic databases, as well as the price information available on the Internet, The annual cost for Vitamin D supplementation in the USA ranges from $10.15 to $47.10 for an adult, and from $13.20 to $20.45 for a child. | Price may vary according to the countries, regions, formulations etc. |
|  | **Is the incremental cost small relative to the net benefits?** | ○ No ○ Probably no **X Uncertain** ○ Probably yes ○ Yes ○ Varies | No cost-effectiveness analysis related to the preventive use of vitamin D for allergic diseases was identified during the literature review. |  |
| Equity | **What would be the impact on health inequities?** | ○ Increased **X Probably increased** ○ Uncertain ○ Probably reduced ○ Reduced ○ Varies | No research evidence | In some settings it may be important to consider equity as the access may depend on socioeconomic status of the country or setting where coverage will depend on policymakers.  It may incur equity problems to invest resources on strategies with “probably no” large desirable effects, rather than invest on other effective treatment/prevention options. |
| Acceptability | **Is the option acceptable to key stakeholders?** | ○ No ○ Probably no ○ Uncertain ○ Probably yes **X Yes** ○ Varies | No research evidence | Vitamin D supplementation is acceptable. However, it could vary according to the setting (i.e., Australia). |
| Feasibility | **Is the option feasible to implement?** | ○ No ○ Probably no ○ Uncertain ○ Probably yes **X Yes** ○ Varies | No research evidence | Vitamin D supplementation is feasible. However, it could vary according to the setting (i.e., Australia). |
|  |  |  |  |  |

| **Recommendation**  **Question: Should VITAMIN D vs. no VITAMIN D be used in infants for prevention of allergies** | | | | | |
| --- | --- | --- | --- | --- | --- |
| **Balance of consequences** | Undesirable consequences *clearly outweigh* desirable consequences in most settings | Undesirable consequences *probably outweigh* desirable consequences in most settings | The balance between desirable and undesirable consequences *is closely balanced or uncertain* | Desirable consequences *probably outweigh* undesirable consequences in most settings | Desirable consequences *clearly outweigh* undesirable consequences in most settings |
|  | ○ | ○ | X | ○ | ○ |

| **Type of recommendation** | We recommend against offering this option | We suggest not offering this option | We suggest offering this option | We recommend offering this option |
| --- | --- | --- | --- | --- |
|  | ○ | X | ○ | ○ |
| **Recommendation** | We suggest that vitamin D supplementation **not be** used in infants (conditional recommendation, very low quality of evidence).  *This recommendation does not apply to infants who have a specific indication for vitamin D supplementation.*  *This will be reviewed and updated as appropriate. Further research will likely have an impact on this recommendation.* | | | |
| **Justification** | Currently there is no evidence suggesting a benefit from supplementation of vitamin D in pregnant women who have a specific indication for vitamin D supplementation. This recommendation is based on very low certainty of evidence, as well as this strategy is not cost-effective and probably increasing health inequities. | | | |
| **Subgroup considerations** | This recommendation for prevention for allergic diseases does not apply to vitamin D deficient women. | | | |
| **Implementation considerations** |  | | | |
| **Monitoring and evaluation** | Incoming studies addressing this research question. | | | |
| **Research possibilities** | RCTs in children. If trails are being done for other reasons we suggest collecting information of allergy outcomes. | | | |
